# Supplementary material for: AtMYBS1 negatively regulates heat tolerance by directly repressing the expression of MAX1 required for strigolactone biosynthesis in Arabidopsis
Source: Plant Commun. 2023 Aug 22;4(6):100675. doi: 10.1016/j.xplc.2023.100675 (PMC10721535; doi:10.1016/j.xplc.2023.100675)
Supplement: Document S1. Supplemental Figures 1–13 and Supplemental Table 1 [file mmc1.pdf]

Plant Communications, Volume *4*

Supplemental information

AtMYBS1 negatively regulates heat tolerance by directly repressing the expression of *MAX1* required for strigolactone biosynthesis in *Arabidopsis*

Xiang Li, Jianhua Lu, Xuling Zhu, Yanqi Dong, Yanli Liu, Shanshan Chu, Erhui Xiong, Xu Zheng, and Yongqing Jiao

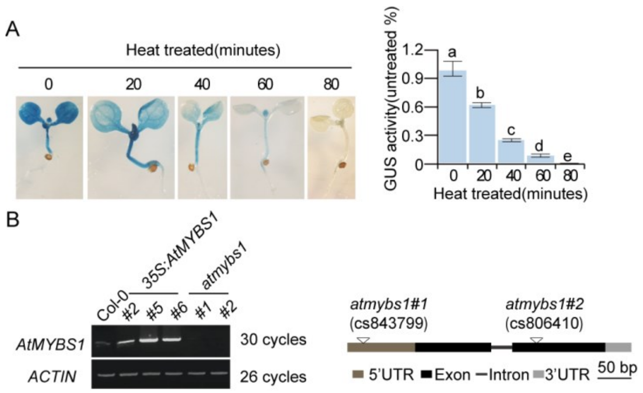

Supplemental Figure 1. Expression pattern of *pAtMYBS1*:*GUS* in response to heat stress and molecular validation of *AtMYBS1* overexpression lines and loss-of-function mutants.

(A) The expression pattern of *pAtMYBS1:GUS* in response to heat stress. Seven-day-old seedlings of *pAtMYBS1: GUS*-positive homozygous transgenic plants grown in half-strength MS medium in a greenhouse (23 °C, 70% humidity, 16 h light/8 h dark cycle) were subjected to heat treatment at 40 °C for the indicated times. Then, GUS staining assays were performed to monitor *AtMYBS1* promoter activity under heat stress conditions. Three independent biological replicates were performed (n>5 for each replicate). The data are the means ± SD. Different letters on error bars indicate significant differences at $P<0.05$, Tukey's *t* test. **(B)** Confirmation of *AtMYBS1* overexpression lines and loss-of-function mutants. Semiquantitative RT-PCRs were performed to measure *AtMYBS1* expression in *AtMYBS1*-overexpressing plants and *atmybs1* mutants. *ACTIN* was used as an internal control. The brown box represents the 5'UTR. The black boxes represent exons, and the black line represents introns. The gray box represents the 3'UTR. Two T-DNA insertion sites are indicated by inverted triangles.

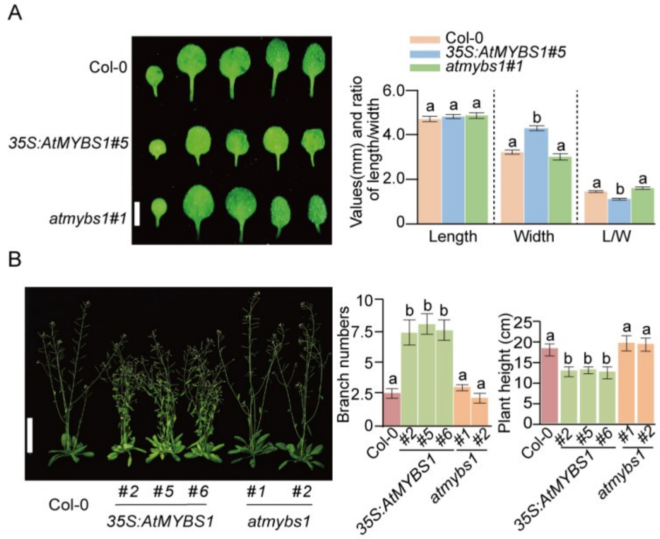

Supplemental Figure 2. Phenotypes of *AtMYBS1*-overexpressing plants and *atmybs1* mutants.
(A) Leaf shape of *AtMYBS1*-overexpressing plants and *atmybs1* mutants. Leaves of 16-day-old plants of Col-0, *35S:AtMYBS1-5* and *atmybs1-1* were collected and photographed; meanwhile, their sizes were measured and documented (n>12 in each sample). L/W: length/width. The data shown are the means ± SD. Different letters on error bars indicate significant differences at $P<0.05$, Tukey's *t* test. Scale bar=5 mm. **(B)** Plant morphologies of *AtMYBS1*-overexpressing plants and *atmybs1* mutants. Branch numbers and plant height from 40-day-old plants of Col-0, *AtMYBS1*-overexpressing lines (*35S:AtMYBS1-2*, *-5* and *-6*) and *atmybs1* (*atmybs1-1* and *-2*) mutants were observed and analyzed (n>10). The data are the means ± SDs. Different letters on error bars indicate significant differences at $P<0.05$, Tukey's *t* test. Scale bar=5 cm.

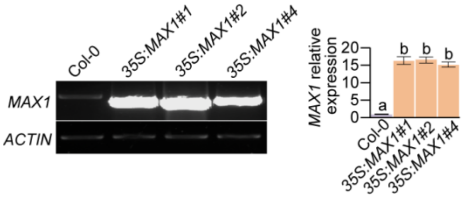

Supplemental Figure 3. The expression of *MAX1* in *MAX1*-overexpressing transgenic lines.
The *35S:MAX1* construct (*pMDC85* vector) was constructed and transformed into wild-type Col-0 plants, and homozygous single copy T-DNA insertion lines were screened and obtained. Seeds of the *35S:MAX1#1, #2,* and *#4* transgenic lines were sown on half-strength MS medium and grown at 23 °C in the greenhouse (16 hours light/8 hours dark, 70% humidity). The 14-day-old plants were harvested for RNA extraction, and semiquantitative RT–PCR was employed to measure *MAX1* expression (left). qRT–PCR was also performed to confirm *MAX1* expression levels (right). Three independent biological replicates were performed. The data are the means ± SDs. Different letters on error bars indicate significant differences at $P<0.05$, Tukey's *t* test.

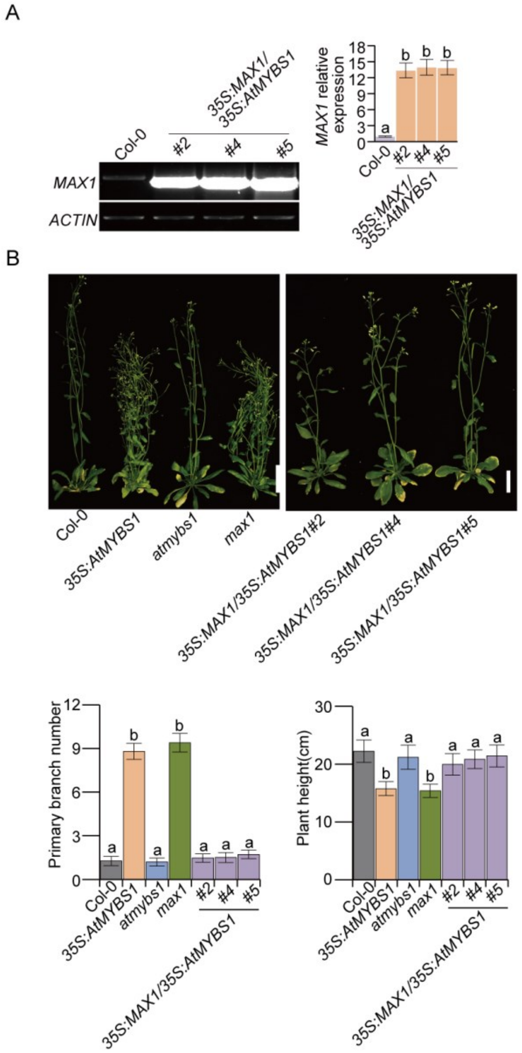

Supplemental Figure 4. Plant morphologies of *35S:MAX1/35S:AtMYBS1* plants.
(A) *MAX1* expression levels in *35S:MAX1/35S:AtMYBS1* plants. The *35S:MAX1* (*pMDC85* vector) construct was transformed into *35S:AtMYBS1-5* plants. T_3 generation plants were screened for homozygous single copy T-DNA insertion lines. Seeds from homozygous lines were sown on half-strength MS medium and grown at 23 °C in the greenhouse (16 hours light/8 hours dark, 70% humidity). The 12-day-old plants were harvested for RNA extraction, and semiquantitative RT–PCR was employed to measure *MAX1* expression (left). qRT–PCR was also performed to confirm the *MAX1* expression level (right). Three independent biological replicates were conducted in the qRT–PCR experiments. The data are the means ± SDs. Different letters on error bars indicate significant differences at P<0.05, Tukey's *t* test. **(B)** Phenotypes of Col-0, *35S:AtMYBS1-5*, *atmybs1-1*, *max1-1* and *35S:MAX1/35S:AtMYBS1-5* transgenic lines. Six-week-old plants of Col-0, *35S:AtMYBS1-5*, *atmybs1-1*, *max1-1* and *35S:MAX1/35S:AtMYBS1-5* transgenic lines (*35S:MAX1/35S:AtMYBS1#2, #4, #5*) grown in a greenhouse were collected and photographed. Their branch numbers and plant height were measured and statistically analyzed (n>10). The data are the means ± SDs. Different letters on error bars indicate significant differences at P<0.05, Tukey's *t* test. Scale bar=4 cm (left), 2 cm (right).

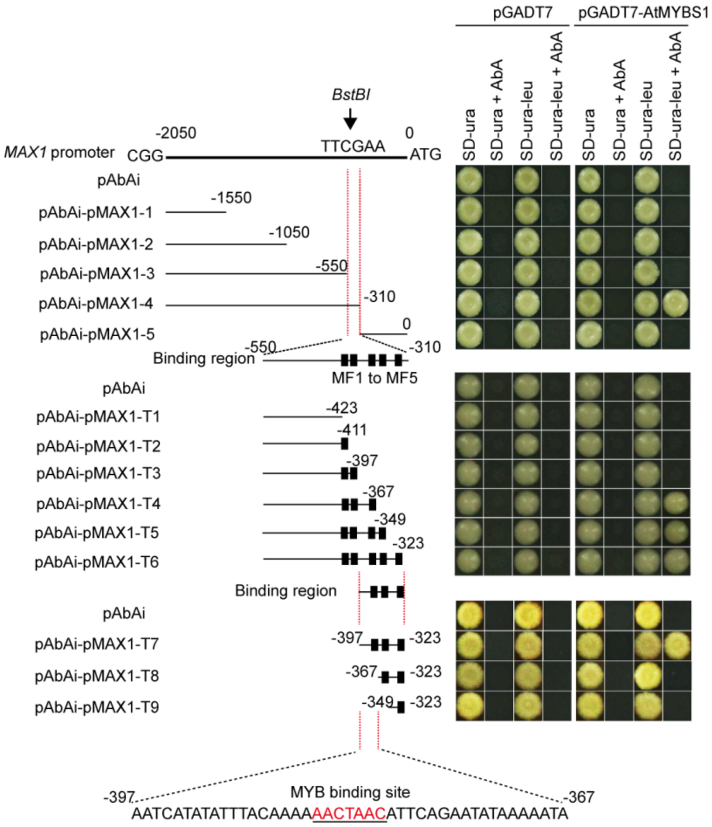

Supplemental Figure 5. The interaction between AtMYBS1 and the *MAX1* promoter detected by yeast one-hybrid assays.

Yeast one-hybrid assays were performed to investigate the interaction between AtMYBS1 and the *MAX1* promoter. The bait vectors (*pAbAi-pMAX1-T1* to *pAbAi-pMAX1-T9*) and the prey vector *pGADT7-AtMYBS1* were constructed and transformed into yeast strain Y1HGold and then plated onto different nutrient-deficient media to examine their interactions. The empty vectors *pAbAi* and *pGADT7* were used as negative controls. All of the solid lines in the picture indicate truncated *MAX1* promoter regions incorporated into *pAbAi*. Numbers indicate exact positions in the *MAX1* promoter. The black boxes represent predictive motifs. The red dotted lines indicate regions to which *AtMYBS1* directly binds according to the yeast one-hybrid results. The underlined red letters indicate MYB-binding sites. Each bait and prey vector was validated by PCR amplification after transformation into the yeast Y1HGold strain. All hybrid assays were repeated at least three times.

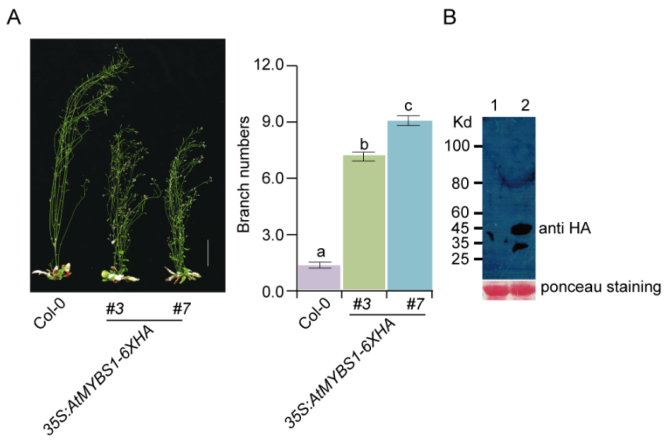

Supplemental Figure 6. Functional confirmation of AtMYBS1-6×HA proteins.

(A) Plant morphologies of HA-tagged *AtMYBS1*-overexpressing transgenic plants. Seven-week-old *35S:AtMYBS1-6×HA* transgenic plants were collected, and their branch numbers were counted (n>15). The data are the means ± SD. Different letters on error bars indicate significant differences at P<0.05, Tukey's *t* test. Scale bar=4 cm. **(B)** Expression of AtMYBS1-6×HA in *35S:AtMYBS1-6×HA* transgenic lines (*35S:AtMYBS1-6×HA*-3 and *35S:AtMYBS1-6×HA*-7) by western blotting. Total proteins were extracted from 12-day-old seedlings of Col-0 and *35S:AtMYBS1-6xHA*-3 and *35S:AtMYBS1-6×HA*-7 transgenic plants. HA antibody was used to detect AtMYBS1-HA expression. Ponceau staining was used as an internal control. The number 1 indicates Col-0; 2 indicates 3*5S:AtMYBS1-6×HA*-7.

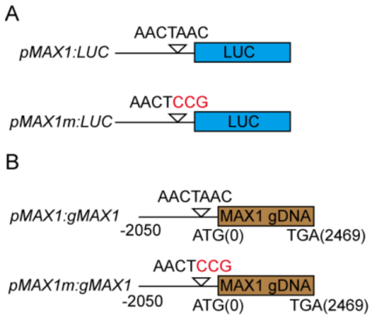

Supplemental Figure 7. Schematic illustrations of constructed vectors.
(**A**) Schematic illustrations of *pMAX1:LUC* and *pMAX1m:LUC* vectors. The black solid lines indicate the *MAX1* promoter. The reverse triangles represent MYB-binding sites. The red letters represent mutated sequences (AAC were changed into CCG). The blue boxes represent luciferase coding sequences. (**B**) Schematic illustrations of *pMAX1:gMAX1* and *pMAX1m:gMAX1* vectors. The black lines indicate the *MAX1* promoter. The inverted triangles represent MYB-binding sites. The red letters indicate mutated nucleotides (AAC were changed into CCG). The brown boxes indicate the *MAX1* genomic sequence after the start codon. Numbers indicate locations corresponding to the *MAX1* genomic sequence.

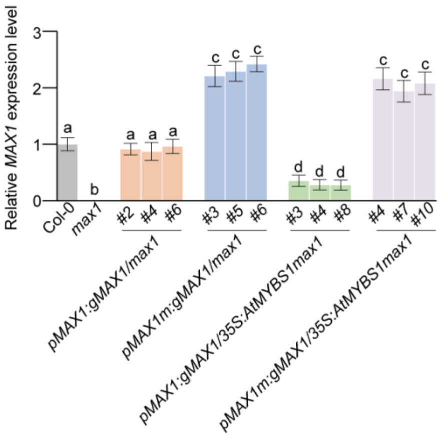

Supplemental Figure 8. ***MAX1*** **expression in** ***pMAX1:gMAX1*** **and** ***pMAX1m:gMAX1*** **transgenic plants in the** ***max1*** **and** ***35S:AtMYBS1max1*** **backgrounds.**

The expression of *MAX1* was measured by qRT–PCR in Col-0, *max1-1*, *pMAX1:gMAX1* transgenic plants in the *max1* background (*pMAX1:gMAX1/max1* #2, #4 and #6), *pMAX1m:gMAX1* transgenic plants in the *max1* background (*pMAX1m:gMAX1/max1* #3, #5 and #6), *pMAX1:gMAX1* transgenic plants in *35S:AtMYBS1max1* (*pMAX1:gMAX1/35S:AtMYBS1max* #3, #4 and #8) and *pMAX1m:gMAX1* transgenic plants in the *35S:AtMYBS1max1* (*pMAX1m:gMAX1/35S:AtMYBS1max1* #4, #7 and #10) background. The *35S:AtMYBS1max1* plants were generated by overexpressing *AtMYBS1* under the 35S promoter in the *max1-1* background. Twelve-day-old seedlings grown on half-strength MS (Murashige and Skoog) medium were harvested for RNA extraction and qRT–PCR analysis to measure *MAX1* expression. Three independent biological replicates were carried out. The data are the means ± SDs. Different letters on error bars indicate significant differences at $P<0.05$, Tukey's *t* test.

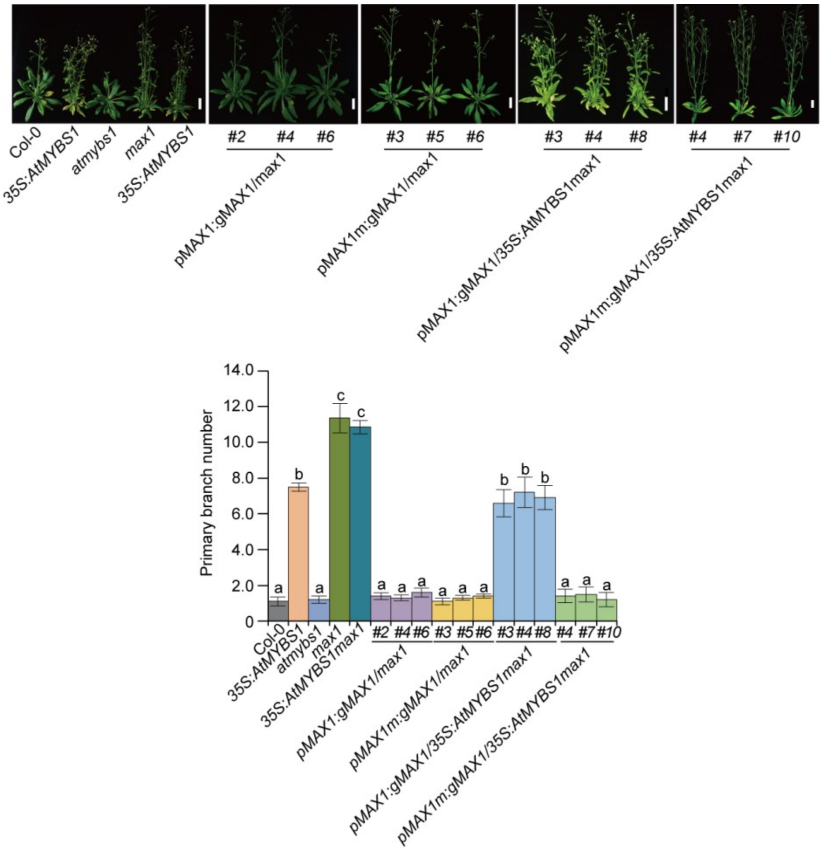

Supplemental Figure 9. Plant morphologies of *pMAX1:gMAX1* and *pMAX1m:gMAX1* transgenic plants in the *max1* and *35S:AtMYBS1max1* backgrounds.

Plant morphologies of Col-0, *35S:AtMYBS1-5, atmybs1-1, max1-1, 35S:AtMYBS1max1-1* #1*, pMAX1:gMAX1* transgenic plants in the *max1* background (*pMAX1:gMAX1/max1* #2, #4 and #6), *pMAX1m:gMAX1* transgenic plants in the *max1* background (*pMAX1m:gMAX1/max1* #3, #5 and #6), *pMAX1:gMAX1* transgenic plants in *35S:AtMYBS1max1* (*pMAX1:gMAX1/35S:AtMYBS1max1* #3, #4 and #8) and *pMAX1m:gMAX1* transgenic plants in the *35S:AtMYBS1max1* (*pMAX1m:gMAX1/35S:AtMYBS1max1* #4, #7 and #10) background. The *35S:AtMYBS1max1* plants were generated by overexpressing *AtMYBS1* under the 35S promoter in the *max1-1* background. All of the above plants were grown in a greenhouse (23 °C, 70% humidity, 16 h light/8 h dark cycle). After 6 weeks, when all the plants had bolted and flowered, different plant samples were harvested, and the number of branches was counted (n>10). The data are the means ± SD. Different letters on error bars indicate significant differences at P<0.05, Tukey's *t* test.

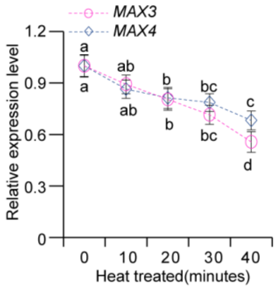

Supplemental Figure 10. ***MAX3*** **and** ***MAX4*** **expression in response to heat stress.**
The wild-type Col-0 seeds sown on half-strength MS medium were grown at 23 °C in the greenhouse (16 hours light/8 hours dark, 70% humidity). The 12-day-old seedlings (two true leaves developed) were treated at 40 °C in a climate chamber (40 °C, 60% humidity, 16 h light/8 h dark cycle) with the time indicated. RNA was extracted from the treated plants after heat treatments, and quantitative RT–PCR was carried out to measure *MAX3* and *MAX4* expression. Three independent biological replications were performed. The data are the means± SDs. Different letters on error bars indicate significant differences at $P<0.05$, Tukey's *t* test.

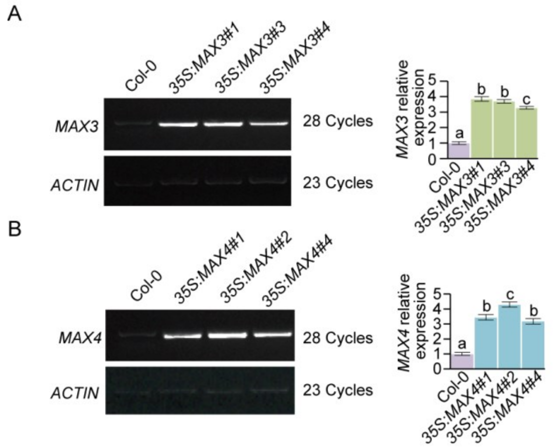

Supplemental Figure 11. Validation of *MAX3* and *MAX4* expression in *35S:MAX3* and *35S:MAX4* plants.

The *35S:MAX3* (*pJL12*) and *35S:MAX4* (*pJL12*) constructs were generated and transformed into wild-type Col-0 plants. Homozygous single copy T-DNA insertion lines were screened and obtained. The seeds of homozygous lines were sown on half-strength MS medium and grown at 23 °C in a greenhouse (16 hours light/8 hours dark, 70% humidity). The 14-day-old plants were harvested for RNA extraction. Subsequent semiquantitative RT–PCR was employed to measure *MAX3* and *MAX4* expression (left). qRT–PCR was also performed to confirm *MAX3* and *MAX4* expression levels **(**right**)**. Three independent biological replicates were conducted. The data are the means ± SDs. Different letters on error bars indicate significant differences at $P<0.05$, Tukey's *t* test.

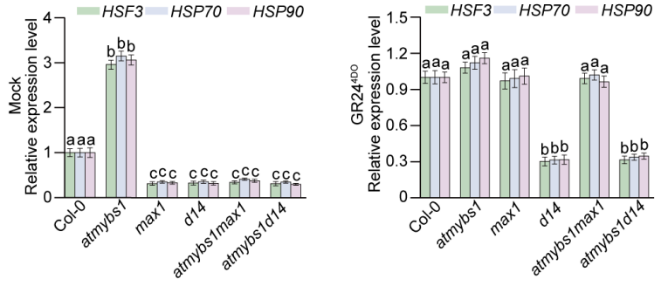

Supplemental Figure 12. Expression of *HSF3, HSP70* and *HSP90* in *max1* mutants, *atmybs1max1* double mutants and *atmybs1d11* double mutants without or with GR24^{4DO} application.
Expression levels of *HSF3*, *HSP70* and *HSP90* were measured by qRT–PCR in Col-0 and atmybs1-1, *max1-1*, *d14-1*, *atmybs1-1max1-1* and *atmybs1-1d14-1* double mutants without or with GR24^{4DO} application. The 14-day-old seedlings grown on half-strength MS medium were collected for RNA extraction. Subsequently, qRT–PCR was performed to measure *HSF3*, *HSP70* and *HSP90* expression. Three independent biological replicates were carried out. The data are the means ± SDs. Different letters on error bars indicate significant differences at $P<0.05$, Tukey's *t* test.

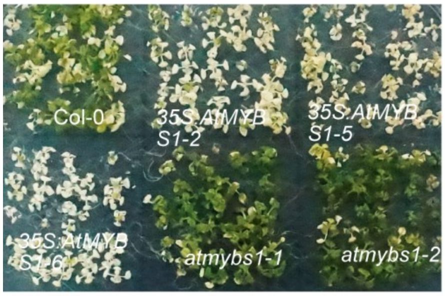

Figure 1B

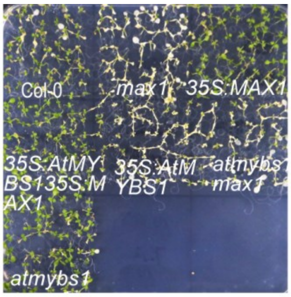

Figure 2C

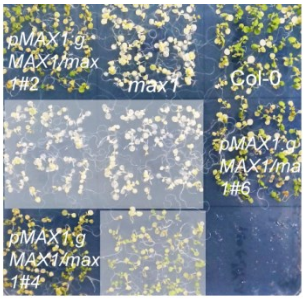

Figure 3D

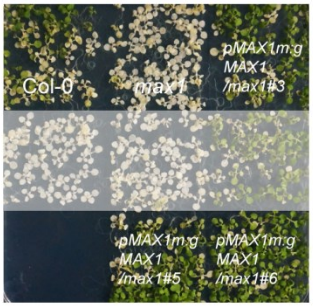

Figure 3D

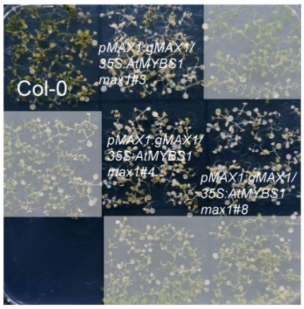

Figure 3E

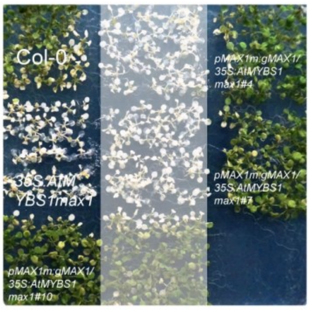

Figure 3E

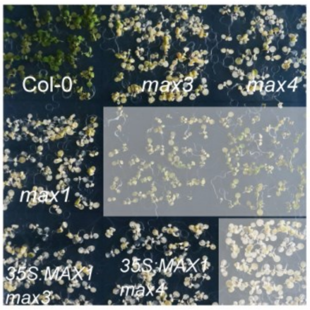

Figure 4A

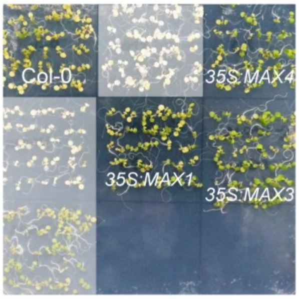

Figure 4A

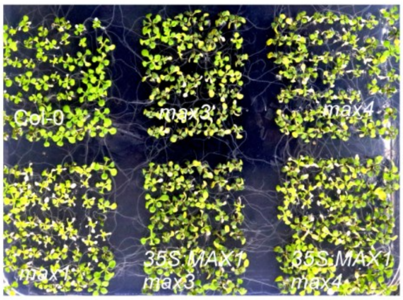

Figure 4B

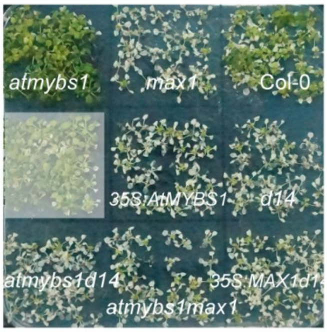

Figure 4C

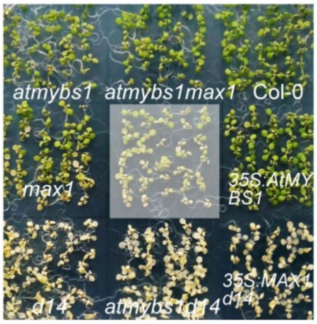

Figure 4C

Supplemental Figure 13. Original photographs for related pictures in this study.
Pictures of the original whole petri dishes used in this study are shown. The samples unrelated to this study are covered by white boxes. The samples used in this study were labeled as indicated.

Supplemental Table 1. Primers used in vector construction and gene expression analyses.

name	sequence	usage
pJL12-AtMYBS1-F	TCTGATCAAGAGACAGATGGAGAGTGTGGTGGCAACATGGAGC	*35S:AtMYBS1*(*pJL12*)
pJL12-AtMYBS1-R	GCTCTAGAACTAGTGTCAGTGCATTGTCGACGGAG	
pAtMYBS1-GUS-F	CAAAAAAGCAGGCTTTGACTTTAGGTC CACCGGACATTTTCTTAAAT	*pAtMYBS1:GUS* (*pKGWFS7*)
pAtMYBS1-GUS-R	AAAGCTGGGTCTAGAGACTTTAGGTC TCGATTGCTTCAATCTTTTC	
pMDC85-MAX1-F	TTCTAGAGTCGAGGTCC ATGAAGACGCAACATCAATG	*35S:MAX1*(*pMDC85*)
pMDC85-MAX1-R	CGGTAGAAAAAATGA TCAGAATCTTTTGATGGTTCTGA	
pGADT7-AtMYBS1-F	ATGGCCATGGAGGCCAGTGAATTC ATGGAGAGTGTGGTGGCAAC	*pGADT7-AtMYB1*
pGADT7-AtMYBS1-R	GGATCCATCGAGCTCGAGCTGCA TCAGTGCATTGTCGACGGAG	
pAbAi-pMAX1-1-F	GCTTGAATTCGAGCT CGGTGATAAACTAATCCACCA	*pAbAi-pMAX1-1*
pAbAi-pMAX1-1-R	AGCACATGCCTCGAGG TTCCTTCCTCCAACAAGATG	
pAbAi-pMAX1-2-F	GCTTGAATTCGAGCT CGGTGATAAACTAATCCACCA	*pAbAi-pMAX1-2*
pAbAi-pMAX1-2-R	AGCACATGCCTCGAGG GGGAGAGACGAAATTTCATC	
pAbAi-pMAX1-3-F	GCTTGAATTCGAGCT CGGTGATAAACTAATCCACCA	*pAbAi-pMAX1-3*
pAbAi-pMAX1-3-R	AGCACATGCCTCGAGG GCTTCTACTTTTGGTCTCCT	
pAbAi-pMAX1-4-F	GCTTGAATTCGAGCT CGGTGATAAACTAATCCACA	*pAbAi-pMAX1-4*
pAbAi-pMAX1-4-R	AGCACATGCCTCGAGG GAAACTATTTAACTATATT	
pAbAi-pMAX1-5-F	GCTTGAATTCGAGCT GAATTAAACACTAAATAATTAAATGTTGAC	*pAbAi-pMAX1-5*
pAbAi-pMAX1-5-R	AGCACATGCCTCGAGG CTCTCTAACCTCTAAAGTTC	
pAbAi-pMAX1-T1-F	GCTTGAATTCGAGCT CTCTCTAACCTCTAAAGTTC	*pAbAi-pMAX1-T1*
pAbAi-pMAX1-T1-R	AGCACATGCCTCGAGG TACTATCTTTTGAGTATGGG	
pAbAi-pMAX1-T2-F	GCTTGAATTCGAGCT GTGGTTTACTCCAATTGACGG	*pAbAi-pMAX1-T2*
pAbAi-pMAX1-T2-R	AGCACATGCCTCGAGG AAATAAACAGAATACTATCT	
pAbAi-pMAX1-T3-F	GCTTGAATTCGAGCT GTGGTTTACTCCAATTGACGG	*pAbAi-pMAX1-T3*
pAbAi-pMAX1-T3-R	AGCACATGCCTCGAGG AAATATATGATTATAAATAA	
pAbAi-pMAX1-T4-F	GCTTGAATTCGAGCT GTGGTTTACTCCAATTGACGGT	*pAbAi-pMAX1-T4*
pAbAi-pMAX1-T4-R	AGCACATGCCTCGAGG ATTTTTATATTCTGAATGTT	
pAbAi-pMAX1-T5-F	GCTTGAATTCGAGCT GTGGTTTACTCCAATTGACGGTAAA	*pAbAi-pMAX1-T5*
pAbAi-pMAX1-T5-R	AGCACATGCCTCGAGG AAATTTACATATTCTAATAT	
pAbAi-pMAX1-T6-F	GCTTGAATTCGAGCT GTGGTTTACTCCAATTGACGG	*pAbAi-pMAX1-T6*
pAbAi-pMAX1-T6-R	AGCACATGCCTCGAGG ATATTTAATACTCCTCTATG	
pAbAi-pMAX1-T7-F	GCTTGAATTCGAGCT TGGTTTACTCCAATTGACGGTAA	*pAbAi-pMAX1-T7*
pAbAi-pMAX1-T7-R	AGCACATGCCTCGAGG ATATTTAATACTCCTCTATG	
pAbAi-pMAX1-T8-F	GCTTGAATTCGAGCT TGACAAGTAGAGGCACTTGATAGTAGTGT	*pAbAi-pMAX1-T8*
pAbAi-pMAX1-T8-R	AGCACATGCCTCGAGG ATATTTAATACTCCTCTATG	
pAbAi-pMAX1-T9-F	GCTTGAATTCGAGCT GATAGTAGTGTAACAAAAGT	*pAbAi-pMAX1-T9*
pAbAi-pMAX1-T9-R	AGCACATGCCTCGAGG ATATTTAATACTCCTCTATG	
pAbAi-pMAX1-T10	GCTTGAATTCGAGCT ACAAAAAACTAACATTCAGAATATAAAAAT CCTCGAGGCATGTGCT	*pAbAi-pMAX1-T10*
pAbAi-pMAX1-MD	GCTTGAATTCGAGCT ACAAAAATTCAGAATATAAAAAT CCTCGAGGCATGTGCT	*pAbAi-pMAX1-MD*

pAbAi-pMAX1-MP	GCTTGAATTCGAGCT ACAAAAAACTCCGATTCAGAATATAAAAT CCTCGAGGCATGTGCT	*pAbAi-pMAX1-MP*
pJL12-AtMYBS1-6×HA-F	GATCTGATCAAGAGACAG ATGGAGAGTGTGGTGGCAACATGGAGC	*35S:AtMYBS1*(*pJL12-6×HA*)
pJL12-AtMYBS1-6×HA-R	GCCCTTGCTCACCATGGA GTGCATTGTCGACGGAG	
pMAX1-pGreenII0800-F	TCGACGGTATCGATA CGGTGATAAACTAATCCACCAA	*pMAX1-pGreenII0800*
pMAX1-pGreenII0800-R	TAGAACTAGTGGATC CTCTCTAACCTCTAAAGTTCTCTCTT	
pMAX1m-pGreenII0800-F1	TCGACGGTATCGATA CGGTGATAAACTAATCCAC	*pMAX1m-pGreenII0800*
pMAX1m-pGreenII0800-R1	CTGAATCGGAGTTTTTTGTAAAT	
pMAX1m-pGreenII0800-F2	TATTTACAAAAAACTCCGATTCAGAAT	
pMAX1m-pGreenII0800-R2	TAGAACTAGTGGATC CTCTCTAACCTCTAAAGTTCTCTCTT	
pMAX1-gMAX1-F	TATGACCATGATTACG CGGTGATAAACTAATCCACCAA	*pMAX1:gMAX1(p1300)*
pMAX1-gMAX1-R	AGGTCGACTCTAGAG TCAGAATCTTTTGATGGTTCTGAG	
pMAX1m-gMAX1-F1	TATGACCATGATTACG CGGTGATAAACTAATCCACCAA	*pMAX1m:gMAX1(p1300)*
pMAX1m-gMAX1-R1	CTGAATCGGAGTTTTTTGTAAAT	
pMAX1m-gMAX1-F2	TATTTACAAAAAACTCCGATTCAGAAT	
pMAX1m-gMAX1-R2	AGGTCGACTCTAGAG TCAGAATCTTTTGATGGTTCTG	
pJL12-MAX3-F	TCTGATCAAGAGACAG ATGTCTCTCCCTATCCCGCCGAA	*35S:MAX3*(*pJL12*)
pJL12-MAX3-R	GCTCTAGAACTAGTG TTATTTGTGTGAAAGGTGAAAAG	
pJL12-MAX4-F	TCTGATCAAGAGACAG ATGGCTTCTTTGATCACAACCAA	*35S:MAX4*(*pJL12*)
pJL12-MAX4-R	GCTCTAGAACTAGTG TTAATCTTTGGGGATCCAGCAAC	
MAX1-qPCR-F	ATGAAGACGCAACATCAATG	
MAX1-qPCR-R	ATCTTTGAACTTCTTTATCC	
MAX2-qPCR-F	ATGGCTTCCACTACTCTCTC	
MAX2-qPCR-R	GTGTGTAGACGTTTAGAGAC	
MAX3-qPCR-F	ATGTCTCTCCCTATCCCGCC	
MAX3-qPCR-R	GGTTCGATAGTCTCGGAACG	
MAX4-qPCR-F	ATGGCTTCTTTGATCACAAC	
MAX4-qPCR-R	TTGTTGTACACTTGTCCACG	
AtMYBS1-qPCR-F	ATGGAGAGTGTGGTGGCAAC	
AtMYBS1-qPCR-R	CCTTTGCGGTGATGATAACG	
ACTIN-qPCR-F	CTCAGCACCTTCCAACAGATGTGGA	
ACTIN-qPCR-R	CCAAAAAAATGAACCAAGGACCAAA	qRT-PCR
HSF3- qPCR-F	ATGAGCCCAAAAAAAGATGCTGTTT	
HSF3- qPCR-R	ATCCCAAACTACGAAGCTAG	
HSP90-qPCR-F	ATGAGGAAGAGGACGCTCGT	
HSP90-qPCR-R	CTCAAACTTCTCCGCGTTAC	
HSP70-qPCR-F	ATGGCGGGTAAAGGTGAAGG	
HSP70-qPCR-R	GAACAGAGGGATCACTGTAT	
R1f	CTCTCTTCTCCACTCTTGCAG	
R1r	CCAGCTCCTAACCCCTTCAC	
R2f	GGTTAAATTGATGAGGTCTC	
R2r	TCAATACATGTAATGCAGAT	
R3f	GTAACTAGATGAAAGAAAAGAA	

R3r	AAAGGAGAAATTTGATCATT	
R4f	TCAAACATCAAAAGGACCCC	
R4r	TTTTTTCTTGTCAACATTTA	
R5f	ATTTACAAAAAACTAACATTCA	
R5r	CGCAAGAAGACAACATGCGT	
R6f	TTAGCAATATTACTCTCTAGTAGA	
R6r	AAAGTTCTCTCTTCTTTAGT	
atmybs1-1-LP	TTATTATTTGCCGCAGTTTCG	*atmybs1-1* genotyping
atmybs1-1-RP	TTACCTCGTTATCATCACCGC	
atmybs1-2-LP	CAAGCTCCTTATCGCGAAAG	*atmybs1-2* genotyping
atmybs1-2-RP	ACCACACTCTCCATTCGATTG	
LB1	GCCTTTTCAGAAATGGATAAATAGCCTTGCTTCC	Universal primer for genotyping

Note: The underlined sequences are adaptors in destiny vectors used in homologous recombination. Brackets indicate destiny vectors that the sequences are ultimately integrated in.
