# Supplementary material for: AtMYBS1 negatively regulates heat tolerance by directly repressing the expression of MAX1 required for strigolactone biosynthesis in Arabidopsis
Source: Plant Commun. 2023 Aug 22;4(6):100675. doi: 10.1016/j.xplc.2023.100675 (PMC10721535; doi:10.1016/j.xplc.2023.100675)
Supplement: Document S2. Article plus supplemental information [file mmc2.pdf]

Plant Communications
Research article

CellPress Partner Journal

AtMYBS1 negatively regulates heat tolerance by directly repressing the expression of *MAX1* required for strigolactone biosynthesis in *Arabidopsis*

Xiang Li[1,2,3], Jianhua Lu[2], Xuling Zhu[1], Yanqi Dong[3], Yanli Liu[3], Shanshan Chu[1], Erhui Xiong[1,*], Xu Zheng[1,*] and Yongqing Jiao[1,2,*]

[1]Collaborative Innovation Center of Henan Grain Crops, College of Agronomy, Henan Agricultural University, Zhengzhou 450002, China

[2]Key Laboratory of Biology and Genetic Improvement of Oil Crops, the Ministry of Agriculture and Rural Affairs, Oil Crops Research Institute of Chinese Academy of Agricultural Sciences, Wuhan 430062, China

[3]Xinxiang Academy of Agricultural Sciences, Xinxiang 453000, China

***Correspondence: Yongqing Jiao (jiaoyongqing@henau.edu.cn), Xu Zheng (zhengxu@henau.edu.cn), Erhui Xiong (xiongerhui@henau.edu.cn)**

https://doi.org/10.1016/j.xplc.2023.100675

ABSTRACT

Heat stress caused by global warming requires the development of thermotolerant crops to sustain yield. It is necessary to understand the molecular mechanisms that underlie heat tolerance in plants. Strigolactones (SLs) are a class of carotenoid-derived phytohormones that regulate plant development and responses to abiotic or biotic stresses. Although SL biosynthesis and signaling processes are well established, genes that directly regulate SL biosynthesis have rarely been reported. Here, we report that the MYB-like transcription factor AtMYBS1/AtMYBL, whose gene expression is repressed by heat stress, functions as a negative regulator of heat tolerance by directly inhibiting SL biosynthesis in *Arabidopsis*. Overexpression of *AtMYBS1* led to heat hypersensitivity, whereas *atmybs1* mutants displayed increased heat tolerance. Expression of *MAX1*, a critical enzyme in SL biosynthesis, was induced by heat stress and downregulated in *AtMYBS1*-overexpression (OE) plants but upregulated in *atmybs1* mutants. Overexpression of *MAX1* in the *AtMYBS1*-OE background reversed the heat hypersensitivity of *AtMYBS1*-OE plants. Loss of *MAX1* function in the *atmyb1* background reversed the heat-tolerant phenotypes of *atmyb1* mutants. Yeast one-hybrid assays, chromatin immunoprecipitation–qPCR, and transgenic analyses demonstrated that AtMYBS1 directly represses *MAX1* expression through the MYB binding site in the *MAX1* promoter *in vivo*. The *atmybs1d14* double mutant, like *d14* mutants, exhibited hypersensitivity to heat stress, indicating the necessary role of SL signaling in *AtMYBS1*-regulated heat tolerance. Our findings provide new insights into the regulatory network of SL biosynthesis, facilitating the breeding of heat-tolerant crops to improve crop production in a warming world.

Key words: *atmybs1*, MAX1, strigolactone, heat, MYB, D14

Li X., Lu J., Zhu X., Dong Y., Liu Y., Chu S., Xiong E., Zheng X., and Jiao Y. (2023). AtMYBS1 negatively regulates heat tolerance by directly repressing the expression of *MAX1* required for strigolactone biosynthesis in *Arabidopsis*. Plant Comm. **4**, 100675.

INTRODUCTION

Extreme high temperature (heat stress) caused by global warming has resulted in devastating damage to crop production (Lobell et al., 2011; Lesk et al., 2016). Development of heat-tolerant crops is therefore urgently needed to secure future food production. To achieve the goal of developing heat-tolerant crops, greater understanding of the molecular mechanisms involved in plant heat tolerance is needed. Strigolactones (SLs) are newly defined phytohormones that play critical roles in regulation of plant architecture (Wang et al., 2018; Burger

Published by the Plant Communications Shanghai Editorial Office in association with Cell Press, an imprint of Elsevier Inc., on behalf of CSPB and CEMPS, CAS.

© 2023 The Authors.
This is an open access article under the CC BY license (http://creativecommons.org/licenses/by/4.0/).

and Chory, 2020) and protection against adverse conditions, including drought and salt stress (Ha et al., 2014; Mostofa et al., 2018), fungal intrusion (Decker et al., 2017), and seed thermoinhibition (Toh et al., 2012). SL biosynthesis requires successive catalytic processes and a series of enzymes, with the first step being isomerization of all-*trans*-β-carotene into 9-*cis*-β-carotene by *DWARF27* (D27) (Alder et al., 2012). 9-*cis*-β-Carotene then undergoes successive catalytic processes catalyzed by carotenoid cleavage dioxygenases 7 and 8 (MAX3 and MAX4), which ultimately produce carlactone (CL) (Alder et al., 2012). CL is transported into the cytoplasm and further oxidized by the cytochrome P450 711A (CYP711A) family to yield carlactonoic acid (CLA) (Mashiguchi et al., 2021). CLA can be transformed into two types of SLs: canonical and noncanonical SLs (Mashiguchi et al., 2021). Canonical SLs have a tricyclic lactone structure composed of three rings (ABC rings) (Boyer et al., 2012; Umehara et al., 2015; Mashiguchi et al., 2021), whereas noncanonical SLs lack typical ABC rings (Xie et al., 2019; Mashiguchi et al., 2021). Some members of the CYP711A and CYP722C families can produce the canonical SLs 4DO, 5DS, and ORO from CLA (Wakabayashi et al., 2020; Mashiguchi et al., 2021). *MAX1* encodes CYP711A1, and loss of its function produces a hyperbranching phenotype (Stirnberg et al., 2002; Booker et al., 2005; Mashiguchi et al., 2021). For noncanonical SLs, methyl carlactonoate (MeCLA) is a key intermediate that can be produced from CLA by a CLA methyltransferase in *Arabidopsis* (Brewer et al., 2016; Waters et al., 2017; Mashiguchi et al., 2022). Exogenous application of CLA or MeCLA can rescue *max1* mutant phenotypes; however, only MeCLA can be perceived by the SL receptor D14 (Abe et al., 2014; Waters et al., 2017; Mashiguchi et al., 2022). After SL synthesis, the receptor D14 recognizes SLs and interacts with the F-box protein MAX2 to form the SKP1–CULLIN–F-BOX (SCF) complex, which degrades downstream substrates (D53, SMXL6, SMXL7, SMXL8, and others) to fulfill SL function (Arite et al., 2009; Shabek et al., 2018; Marzec and Brewer, 2019).

MYB proteins are characterized by a highly conserved DNA-binding domain called the MYB domain. This domain generally consists of up to four amino acid sequence repeats (R) of approximately 52 amino acids (Dubos et al., 2010). MYB proteins can be categorized into different subfamilies according to the number of repeats. Plant MYB proteins are divided into four major groups: R2R3-MYB, with two adjacent repeats; R1R2R3-MYB (3R-MYB), with three adjacent repeats; R1R2R2R1/2-MYB (4R-MYB), with four adjacent repeats; and R1/2-MYB, a group of heterogeneous MYB-like (MYBL) proteins that usually but not always contain a single MYB repeat (Dubos et al., 2010). The majority of MYB-family proteins function as transcription factors to affect various aspects of plant growth and responses to biotic and abiotic stresses (Dubos et al., 2010). The *AtMYBS1/AtMYBL* gene encodes an R1/2-MYB-like protein that was first reported to modulate leaf senescence and the response to abscisic acid (ABA) and salt stress (Zhang et al., 2011). Overexpression of *AtMYBS1/AtMYBL* enhanced leaf senescence but reduced salt tolerance (Zhang et al., 2011). It was then shown to participate in sugar signaling, similar to its rice homolog *OsMYBS1* (Lu et al., 2002; Chen et al., 2017). *AtMYBS1/AtMYBL* loss-of-function mutants exhibited hypersensitivity to sugars and increased expression of sugar-responsive genes, including genes encoding hexokinase (*HXK1*), chlorophyll *a/b*-binding protein (*CAB1*), and ADP-glucose pyrophosphorylase (*APL3*) (Lu et al., 2002). In addition to the sugar pathway, the ABA pathway is also an important pathway regulated by *AtMYBS1*. Downregulation or loss of function of *AtMYBS1* in *Arabidopsis* results in hypersensitivity to ABA, whereas overexpression of *AtMYBS1* causes a reduced response to ABA (Zhang et al., 2011; Chen et al., 2017).

In this study, we demonstrated that AtMYBS1/AtMYBL plays a negative regulatory role in plant heat tolerance by directly inhibiting expression of *MAX1*, which encodes a critical enzyme in SL biosynthesis. Heat tolerance regulated by *AtMYBS1–MAX1* was also found to depend on SL signaling pathways. Our findings provide new insights into the regulatory network of the SL pathway.

RESULTS

AtMYBS1 is a negative regulator of heat tolerance in *Arabidopsis*

In studies of differentially expressed genes in response to heat stress in *Arabidopsis*, we found that the MYB-like gene *AtMYBS1/AtMYBL* (*At1g49010*) was significantly downregulated during heat treatment (Figure 1A). To confirm the expression pattern of *AtMYBS1*, we generated transgenic plants harboring the β-glucuronidase (GUS) reporter gene driven by the *AtMYBS1* promoter. A GUS activity assay demonstrated a clear reduction in *AtMYBS1* expression in response to heat treatment (Supplemental Figure 1A).

To investigate the function of *AtMYBS1*, we generated *AtMYBS1*-overexpressing (OE) lines (*35S:MYBS1*) by overexpressing *AtMYBS1* driven by the 35S promoter (Supplemental Figure 1B). We also ordered two T-DNA insertion mutants, *CS843799* and *CS806410*, from the SALK mutant collections, which we designated *atmybs1-1* and *atmybs1-2*, respectively (Supplemental Figure 1B). Both of these mutants were null alleles (Supplemental Figure 1B). Phenotypic analyses showed that *AtMYBS1*-OE plants exhibited hypersensitivity to heat stress compared with wild-type Columbia-0 (Col-0) plants (Figure 1B). By contrast, *atmybs1* mutants were more resistant to heat stress (Figure 1B). Consistent with these phenotypic changes, the heat-stress-responsive genes *HSF3*, *HSP70*, and *HSP90* were downregulated in *AtMYBS1*-OE plants but upregulated in *atmybs1* mutants (Figure 1C). We therefore concluded that *AtMYBS1* was a negative regulator of plant heat tolerance.

In addition to their heat-tolerant phenotypes, *AtMYBS1*-OE plants also had rounder and lighter green leaves (Supplemental Figure 2A), increased branching, and reduced plant height compared with Col-0 plants (Supplemental Figure 2B). The *atmybs1* mutants had no significant differences in plant morphology from the Col-0 controls (Supplemental Figure 2A and 2B). Because of similar phenotypes among the different lines, we chose *35S:AtMYBS1#5* and *atmybs1#1* to represent *AtMYBS1*-OE plants and *atmybs1* mutants in subsequent studies.

AtMYBS1 negatively regulates *MAX1* expression in the regulation of heat tolerance

Based on phenotypic similarities between *AtMYBS1-OE plants and SL-related mutants* (e.g., dwarf and bushy architecture and rounder and lighter green leaves) (Stirnberg et al., 2002; Waters

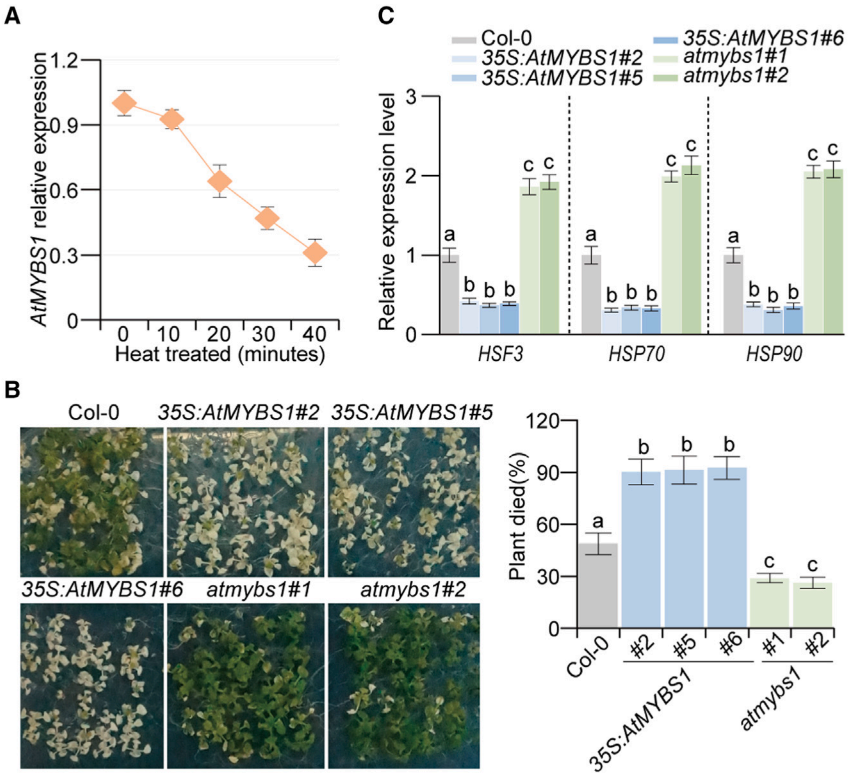

Figure 1. *AtMYBS1* negatively regulates plant heat tolerance.

(A) Expression patterns of *AtMYBS1* in response to heat treatment. Seedlings of wild-type Col-0 grown on half-strength Murashige and Skoog (MS) medium for 14 days were exposed to high temperature in a climate chamber (40°C, 60% humidity, 16 h light/8 h dark cycle) for different times as indicated. At the end of the treatment, plants were collected for RNA extraction, and qRT−PCR was performed to measure *AtMYBS1* expression. Three independent biological replicates were performed. Data are means ± SD.

(B) Heat tolerances of Col-0 and *AtMYBS1*-overexpressing plants and *atmybs1* mutants. Seedlings of Col-0, *AtMYBS1*-overexpressing lines (*35S:AtMYBS1*-2, -5, and -6), and *atmybs1* mutant alleles (*atmybs1*-1 and -2) grown on half-strength MS plates for 14 days in the greenhouse (23°C, 70% humidity, 16 h light/8 h dark cycle) were subjected to heat treatment at 40°C for 6 h in a climate chamber and then recovered at 23°C for 2 h. For survival analysis, plants whose shoot apices turned white were deemed dead. Three biological replicates were performed (n > 50 for each replicate). Data are means ± SD; different letters on error bars indicate significant differences at $P < 0.05$, Tukey's *t*-test.

(C) Expression of the heat-responsive genes *HSF3*, *Hsp70*, and *Hsp90* in *AtMYBS1*-overexpressing lines and *atmybs1* mutants. Ten-day-old seedlings of Col-0, *AtMYBS1*-overexpressing lines (*35S:AtMYBS1*-2, -5, and -6), and *atmybs1* mutants (*atmybs1*-1 and -2) grown on half-strength MS plates in a greenhouse (23°C, 70% humidity, 16 h light/8 h dark cycle) were collected for RNA extraction, and qRT−PCR was performed to measure *HSF3*, *HSP70*, and *HSP90* expression. Three independent biological replicates were performed. Data are means ± SD; different letters on error bars indicate significant differences at $P < 0.05$, Tukey's *t*-test.

et al., 2012a), we speculated that overexpression of *AtMYBS1* might inhibit the SL pathway. To investigate this hypothesis, we examined the expression of four genes involved in the SL pathway, *MAX1* to *MAX4*, in *AtMYBS1*-OE plants and *atmybs1* mutants. The results showed that *MAX1* expression was reduced (∼2-fold) in *AtMYBS1*-OE plants but increased (∼2.5-fold) in *atmybs1* mutants (Figure 2A). *MAX2* expression did not differ among these samples (Figure 2A). *MAX3* and *MAX4* expression was significantly increased (∼4-fold) in *AtMYBS1*-OE plants but slightly decreased in *atmybs1* mutants (Figure 2A). Previous studies found that *MAX3* and *MAX4* were upregulated in *max1* and *max2* mutants, which might be attributed to negative feedback regulation when SL signaling was suppressed (Bennett et al., 2006; Stirnberg et al., 2007; Brewer et al., 2009; Hayward et al., 2009; Waters et al., 2012b). Based on the above findings, we concluded that *AtMYBS1* negatively regulates *MAX1* expression *in vivo*.

To investigate the role of *MAX1* in *AtMYBS1*-regulated heat tolerance, we first examined the pattern of *MAX1* expression under heat treatment. The results showed that *MAX1* was continuously upregulated during heat treatment (Figure 2B), which was opposite to the *AtMYBS1* pattern (Figure 1A and Supplemental Figure 1A). Second, we evaluated the heat tolerance of *MAX1* loss-of-function mutants and *MAX1*-OE transgenic plants (*35S:MAX1*). The results showed that *max1* mutants were sensitive to heat stress, but *MAX1*-OE plants were tolerant (Figure 2C and Supplemental Figure 3). Third, we overexpressed *MAX1* in the *AtMYBS1*-OE background (*35S:MAX1*/*35S:MYBS1*) and found that *MAX1* overexpression (*35S:MAX1*/*35S:MYBS1*) reversed the heat-sensitive phenotypes of *AtMYBS1*-OE plants (Figure 2C and Supplemental Figure 4A). In addition to changes in heat tolerance, overexpression of *MAX1* in the *AtMYBS1*-OE background also reversed the dwarf and excessive branching phenotypes of *AtMYBS1*-OE plants (Supplemental Figure 4B). Finally, we generated *atmybs1max1* double mutants and found that loss of function of *MAX1* in the *atmybs1* background reversed the heat-tolerant phenotypes of *atmybs1* mutants (Figure 2C). In summary, we concluded that *MAX1* expression was negatively regulated by *AtMYBS1* and that *MAX1* participated in the regulation of heat tolerance by *AtMYBS1*.

AtMYBS1 can directly regulate *MAX1* through the MYB binding site in the *MAX1* promoter

To investigate whether *MAX1* was the direct target of *AtMYBS1*, we first performed yeast one-hybrid assays to examine whether AtMYBS1 can bind the *MAX1* promoter *in vitro*. Five truncated *MAX1* promoter segments from −2050 to ATG (pMAX1-1 to pMAX1-5) were constructed and examined (Supplemental Figure 5). The results showed that AtMYBS1 could bind the region from −550 bp to −310 bp in the *MAX1* promoter (Supplemental Figure 5). Five motifs (MF1 to MF5) were detected in this region (Supplemental Figure 5). To determine which motif interacts with AtMYBS1, we performed assays for nine segments (pMAX1-T1 to pMAX1-T9) with different truncations from −550 bp to −310 bp in the *MAX1* promoter. The results

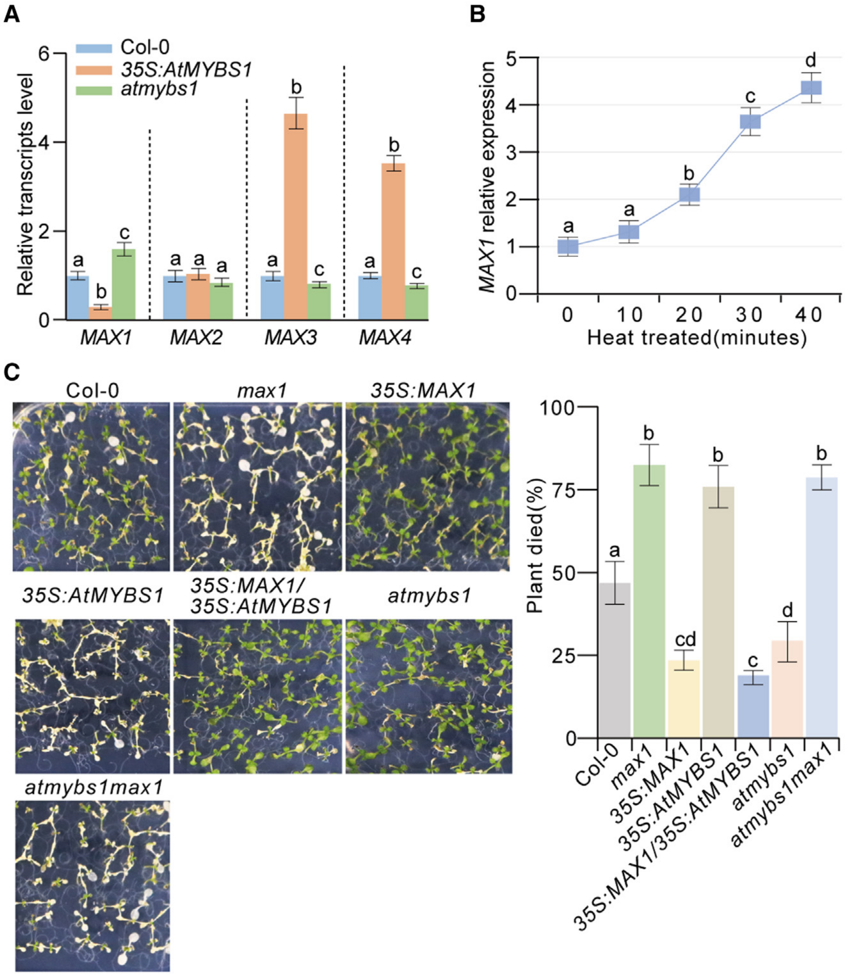

Figure 2. *MAX1* is negatively regulated by AtMYBS1 during the regulation of heat tolerance.

(A) *MAX1–MAX4* expression levels in Col-0, *AtMYBS1*-overexpressing plants, and *atmybs1* mutants measured by qRT–PCR. Twelve-day-old seedlings of Col-0, the *AtMYBS1*-overexpressing line *35S:AtMYBS1-5*, and the *atmybs1* mutant *atmybs1-1* grown on half-strength MS plates were collected for RNA extraction, and qRT–PCR was performed to measure *MAX1–MAX4* expression. Three independent biological replicates were performed. Data are means ± SD; different letters on error bars indicate significant differences at $P < 0.05$, Tukey's *t*-test.

(B) *MAX1* expression pattern during heat treatment. Ten-day-old seedlings of Col-0 grown in the greenhouse (23°C, 70% humidity, 16 h light/8 h dark cycle) were subjected to heat treatment at 40°C for different times as indicated. qRT–PCR was used to measure *MAX1* expression. Three independent biological replicates were performed. Data are means ± SD; different letters on error bars indicate significant differences at $P < 0.05$, Tukey's *t*-test.

(C) Heat tolerance of *MAX1*- and *AtMYBS1*-related plants. Fourteen-day-old seedlings of Col-0, *max1-1* mutants, *35S:MAX1#1*, *35S:MAX1/35S:AtMYBS1-5#2*, *35S:AtMYBS1-5*, *atmybs1-1*, and *atmybs1max1-1* double mutants grown in half-strength MS medium in the greenhouse (23°C, 70% humidity, 16 h light/8 h dark cycle) were treated at 40°C for 6 h in a climate chamber and then recovered at 23°C for 2 h in the greenhouse. Plants whose shoot apices turned white were deemed dead. The plant death rates were calculated and statistically analyzed after treatment. Three independent biological replicates were performed (n > 50 plants for each replicate). Data are means ± SD; different letters on error bars indicate significant differences at $P < 0.05$, Tukey's *t*-test.

showed that the MF3 motif between −397 bp and −367 bp was responsible for the interaction (Supplemental Figure 5). Sequence analyses identified an MYB binding site (AACTAAC) in the MF3 motif (Figure 3A and Supplemental Figure 5). To determine whether this MYB binding site was required for the interaction, we deleted it (pMAX1-MD) or introduced an AACTAAC to AACTCCG mutation (pMAX1-MP) and found that the interaction disappeared (Figure 3A). This result confirmed the necessary role of the MYB binding site in the interaction between AtMYBS1 and *MAX1 in vitro*.

To confirm that AtMYBS1 can directly regulate *MAX1 in vivo*, we performed chromatin immunoprecipitation (ChIP)–qPCR experiments with *35S:AtMYBS1*-6×*HA* transgenic plants. The hemagglutinin (HA)-tagged transgenic lines also displayed an increased branching phenotype, indicating that AtMYBS1-6×HA functioned normally (Supplemental Figure 6). The ChIP–qPCR results showed that the regions between R4 and R5 encompassing the MYB binding site were significantly enriched (Figure 3B), confirming that AtMYBS1 directly binds the *MAX1* promoter through the MYB binding site *in vivo*. We also performed luciferase (LUC) reporter gene assays in *Nicotiana benthamiana* to examine the transcriptional repression of *MAX1* by AtMYBS1. Two different *MAX1* promoters with native (AACTAAC) or mutated (AACTCCG) MYB binding sites were used to drive the expression of luciferase (*pMAX1:LUC* and *pMAX1m:LUC*) (Supplemental Figure 7A). The assays showed that LUC signals were significantly repressed by AtMYBS1 when AtMYBS1 and *pMAX1:LUC* were co-expressed (Figure 3C), but this repression was lost when AtMYBS1 and *pMAX1m:LUC* were co-expressed (Figure 3C). These results confirmed the transcriptional repression of *MAX1 by* AtMYBS1 and the role of the MYB binding site in this repression.

To investigate whether the MYB binding site functioned in the regulation of heat tolerance *in vivo*, we constructed two vectors, *pMAX1:gMAX1* and *pMAX1m:gMAX1*, in which *MAX1* promoters with native (AACTAAC) and mutated (AACTCCG) MYB binding sites were used to drive *MAX1* expression (Supplemental Figure 7B). We first transformed these two vectors into *max1* mutants (*pMAX1:gMAX1/max1* and *pMAX1m:gMAX1/max1*) and evaluated the heat tolerance of the transgenic plants. In *pMAX1:gMAX1/max1* plants, *MAX1* expression was similar to that in wild-type Col-0 (Supplemental Figure 8), and the transgenic plants displayed no significant difference in heat tolerance from Col-0 (Figure 3D). By contrast, *MAX1* expression was significantly enhanced in *pMAX1m:gMAX1/max1 plants* (Supplemental Figure 8), and the transgenic plants exhibited greater heat tolerance than Col-0 (Figure 3D).

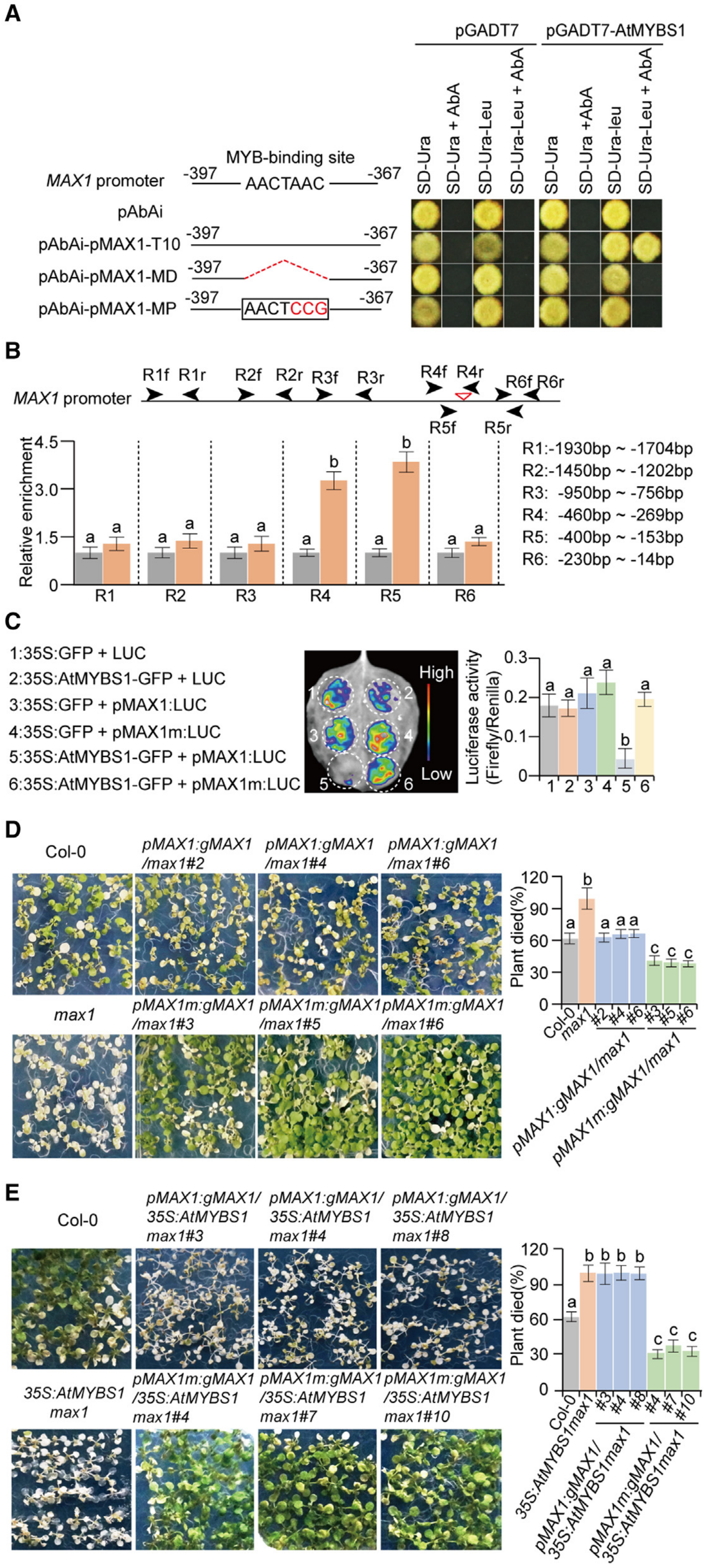

Figure 3. *MAX1* is directly targeted by *AtMYBS1* through the MYB binding site in the *MAX1* promoter.

(A) The interaction between AtMYBS1 and the MYB binding site in the *MAX1* promoter was detected by yeast one-hybrid assay. Three bait vectors, pAbAi-pMAX1-T10 (the region from −397 to −367 bp in the *MAX1* promoter), pAbAi-pMAX1-MD (MYB binding site deleted), and pAbAi-pMAX1-MP (AAC to CCG in the MYB binding site), plus the prey vector pGADT7-AtMYBS1 were co-transformed into yeast strain Y1H Gold and then plated onto specific nutrient-deficient media to test the interactions between AtMYBS1 and the *MAX1* promoter. The empty vectors pAbAi and pGADT7 were used as negative controls. The black solid lines indicate sequences incorporated into *pAbAi* in the *MAX1* promoter. The red dotted lines represent deleted sequences. The red letters indicate replaced nucleotides. The numbers indicate the positions of integrated segments corresponding to the *MAX1* promoter.

(B) Confirmation of the interaction between AtMYBS1 and the MYB binding site *in vivo* by chromatin immunoprecipitation (ChIP)–qPCR. ChIP was performed with HA-tagged *AtMYBS1*-overexpressing plants (*35S:AtMYBS1-6XHA*) using an HA antibody. Primer pairs (R1f, R1r to R6f, R6r) around the MYB binding site were designed to verify the interaction between AtMYBS1 and the MYB binding site. Accurate locations of primers in the *MAX1* promoter are labeled on the right. The inverted triangle represents the AtMYBS1 binding site. Three independent biological replicates were performed. Data are means ± SD; different letters on error bars indicate significant differences at $P < 0.05$, Tukey's *t*-test.

(C) Transcriptional repression activity of AtMYBS1 determined by luciferase (LUC) reporter gene assays in *Nicotiana benthamiana* leaf cells. *35S:AtMYBS1-GFP*, *pMAX1:LUC*, and *pMAX1m:LUC* vectors were constructed and co-transformed into *N. benthamiana* leaf cells. The empty vectors *35S:GFP* and LUC (*pGreenII0800*) were used as internal controls. Three independent biological replicates were performed with similar results. Data are means ± SD; different letters on error bars indicate significant differences at $P < 0.05$, Tukey's *t*-test.

(D) Functions of *MAX1* and MYB binding sites in the regulation of heat tolerance. Fourteen-day-old seedlings of Col-0, *max1-1* mutants, *pMAX1:gMAX1/max1* transgenic plants (#2, #4, #6), and *pMAX1m:gMAX1/max1* transgenic plants (#3, #5, #6) grown in half-strength MS medium in the greenhouse (23°C, 70% humidity, 16 h light/8 h dark cycle) were treated at 40°C for 6 h in a climate chamber, then recovered at 23°C for 2 h in the greenhouse. For survival rate analysis, seedlings whose leaves and shoot apices turned white were deemed dead. Three independent biological replicates were performed ($n > 50$ for each replicate). Data are means ± SD; different letters on error bars indicate significant differences at $P < 0.05$, Tukey's *t*-test.

(E) Roles of *MAX1* and MYB binding sites in *AtMYBS1*-regulated plant heat tolerance. Fourteen-day-old seedlings of Col-0, *35S:AtMYBS1max1-1#1*, *pMAX1:gMAX1/35S:AtMYBS1max1* (#3, #4, #8), and *pMAX1m:gMAX1/35S:AtMYBS1max1* (#4, #7, #10) were subjected to heat treatment as described in **(C)**. Death rates were calculated as described in **(C)**. Three independent biological replicates were performed ($n > 50$ for each replicate). Data are means ± SD; different letters on error bars indicate significant differences at $P < 0.05$, Tukey's *t*-test.

Both *pMAX1:gMAX1/max1* and *pMAX1m:gMAX1/max1* plants exhibited decreased branching phenotypes compared with *max1* mutants, which were similar to those of Col-0 (Supplemental Figure 9). These results confirmed that the MYB binding site in the *MAX1* promoter plays a role in the regulation of heat tolerance *in vivo*.

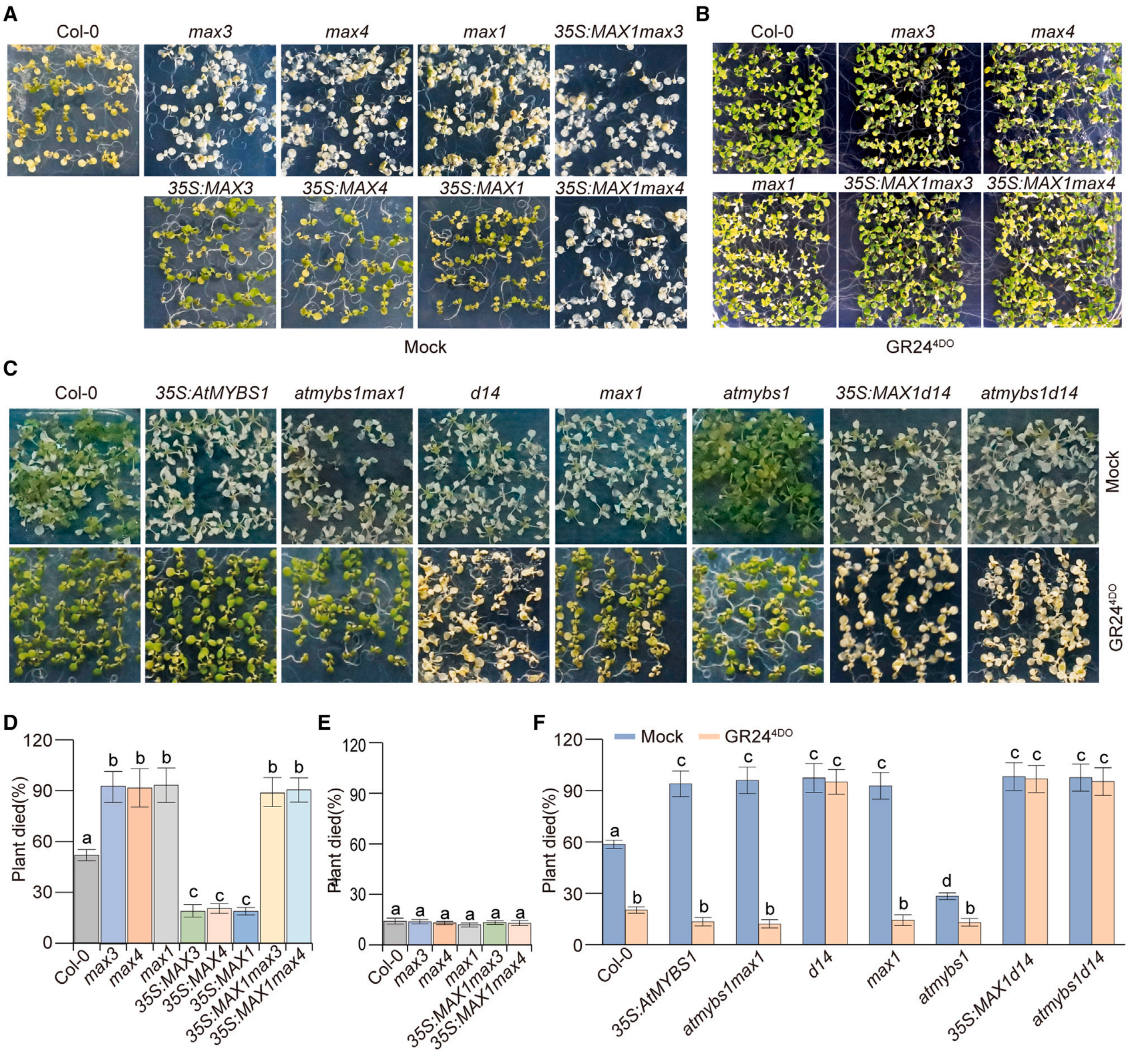

Figure 4. Regulation of heat tolerance by *AtMYBS1* depends on SL biosynthesis and signaling pathways.

(A) Heat tolerance of SL biosynthesis gene mutants and transgenic plants. Twelve-day-old seedlings of Col-0, *max3-9*, *max4-1*, *max1-1*, *35S:MAX1-max3-9#1*, *35S:MAX3#1*, *35S:MAX4#1*, *35S:MAX1#1*, and *35S:MAX1max4-1#1* plants grown in half-strength MS medium in the greenhouse (23°C, 70% humidity, 16 h light/8 h dark cycle) were treated at 40°C for 6 h in a climate chamber (40°C, 60% humidity, 16 h light/8 h dark cycle) and then recovered at 23°C for 2 h in the greenhouse. Photos were taken after treatment. Three independent biological replicates were performed.

(B) Effect of GR24^{4DO} application on the heat tolerance of SL biosynthesis gene mutants and transgenic plants. Twelve-day-old seedlings of Col-0, *max3-9*, *max4-1*, *max1-1*, *35S:MAX1max3-9#1*, and *35S:MAX1max4-1#1* grown in half-strength MS medium in the greenhouse (23°C, 70% humidity, 16 h light/8 h dark cycle) were treated at 40°C for 6 h in a climate chamber (40°C, 60% humidity, 16 h light/8 h dark cycle) and then recovered at 23°C for 2 h in the greenhouse. Photos were taken after treatment. Three independent biological replicates were performed.

(C) AtMYBS1 regulates heat tolerance by repressing *MAX1* and the SL signaling pathway. Twelve-day-old seedlings of Col-0, *35S:AtMYBS1-5*, *atmybs1-1max1-1*, *d14-1*, *max1-1*, *atmybs1-1*, *35S:MAX1d14-1#1*, and *atmybs1-1d14-1* plants grown in half-strength MS medium in the greenhouse (23°C, 70% humidity, 16 h light/8 h dark cycle) were treated at 40°C for 6 h in a climate chamber (40°C, 60% humidity, 16 h light/8 h dark cycle) and then recovered at 23°C for 2 h in the greenhouse. Photos were taken after treatment. Three independent biological replicates were performed.

(D) Statistical analysis of survival rates for the plants in **(A)**. After heat treatment, dead plants were counted and statistically analyzed. The plants whose shoot apices turned white were considered dead. Three independent biological replicates were performed ($n > 50$ for each replicate). Data are means ± SD; different letters on error bars indicate significant differences at $P < 0.05$, Tukey's *t*-test.

(legend continued on next page)

To further examine whether the MYB binding site was responsible for *AtMYBS1*-regulated heat tolerance *in vivo*, we transformed the *pMAX1*:*gMAX1* and *pMAX1m*:*gMAX1* vectors into the *35S*:*AtMYBS1max1* background to generate *pMAX1*:*gMAX1*/*35S*:*AtMYBS1max1* and *pMAX1m*:*gMAX1*/*35S*:*AtMYBS1max1* transgenic plants. *35S*:*AtMYBS1max1* plants were generated by overexpressing *AtMYBS1* under the control of the 35S promoter in the *max1* background. In *pMAX1*:*gMAX1*/*35S*:*AtMYBS1max1* plants, *MAX1* expression was still suppressed (Supplemental Figure 8); the transgenic plants showed heat hypersensitivity and did not differ from *35S*:*AtMYBS1max1* plants in heat sensitivity (Figure 3E). By contrast, *MAX1 expression was significantly enhanced* in *pMAX1m*:*gMAX1*/*35S*:*AtMYBS1max1* plants (Supplemental Figure 8); the transgenic plants exhibited more heat tolerance than *35S*:*AtMYBS1max1* plants and even more than Col-0 (Figure 3E). In addition to changes in heat tolerance, *pMAX1*:*gMAX1*/*35S*:*AtMYBS1max1* plants still displayed increased branching phenotypes similar to those of *AtMYBS1*-OE plants (Supplemental Figure 9). By contrast, *pMAX1m*:*gMAX1*/*35S*:*AtMYBS1max1* plants exhibited reduced branching similar to that of Col-0 (Supplemental Figure 9). In summary, we concluded that the MYB binding site in the *MAX1* promoter was required for regulation of heat tolerance by *AtMYBS1*–*MAX1 in vivo*.

AtMYBS1 regulation of heat tolerance depends on SL signaling pathways

MAX1 was found to be a critical enzyme in SL biosynthesis (Al-Babili and Bouwmeester, 2015). To investigate whether the *AtMYBS1*–*MAX1* module mediates the regulation of heat tolerance through SL biosynthesis, we first analyzed the role of the SL biosynthesis pathway in regulation of heat tolerance *in vivo*. In addition to *MAX1*, we also investigated two other SL biosynthesis genes, *MAX3* and *MAX4*. Gene expression analyses showed that *MAX3* and *MAX4* exhibited slightly decreased expression in response to heat stress (Supplemental Figure 10). Results of heat treatment showed that, like the *max1 mutants,* the *max3* and *max4* mutants were hypersensitive to heat stress (Figure 4A and 4D). Overexpression of *MAX3* and *MAX4* (*35S*:*MAX3* and *35S*:*MAX4*) conferred heat tolerance (Figure 4A and 4D; Supplemental Figure 11), similar to overexpression of *MAX1* (*35S*:*MAX1*). We also generated *35S*:*MAX1*/*max3* and *35S*:*MAX1*/*max4* plants in which *MAX1* was overexpressed in the *max3* and *max4* backgrounds. We found that *35S*:*MAX1*/*max3* and *35S*:*MAX1*/*max4* plants still exhibited heat hypersensitivity similar to that of the *max3* and *max4* mutants (Figure 4A and 4D). Application of the SL analog $GR24^{4DO}$ reversed the heat-hypersensitive phenotypes of the *max1*, *max3*, *max4*, *35S*:*MAX1*/*max3*, and *35S*:*MAX1*/*max4* plants (Figure 4B and 4E). Application of $GR24^{4DO}$ also reversed the heat-hypersensitive phenotypes of *AtMYBS1*-OE and *atmybs1max1* plants (Figure 4C and 4F). In addition, *MAX1* overexpression in the *AtMYBS1*-OE background (*35S*:*MAX1*/*35S*:*AtMYBS1*) reversed the heat-sensitive phenotypes of *AtMYBS1*-OE plants, and loss of function of *MAX1* in the *atmybs1* background (*atmybs1max1* double mutants) reversed the heat-tolerant phenotypes of *atmybs1* mutants (Figure 2C). These results indicated that the SL biosynthesis pathway played a positive role in the regulation of heat tolerance and was also required for *AtMYBS1*–*MAX1*-mediated regulation of heat tolerance.

To determine whether the regulation of heat tolerance by *AtMYBS1* occurred through the SL signaling pathway, we first evaluated the heat tolerance of SL receptor *d14* mutants (Burger and Chory, 2020; Mashiguchi et al., 2021). The results showed that *d14* mutants were hypersensitive to heat stress (Figure 4C and 4F), and $GR24^{4DO}$ application could not reverse this hypersensitivity (Figure 4C and 4F). Loss of function of *D14* in the *35S*:*MAX1* background (*35S*:*MAX1d14*) reversed the heat-tolerant phenotypes of *35S*:*MAX1* plants and caused heat hypersensitivity similar to that of *d14* mutants (Figure 4C and 4F). These results indicated that SL signaling pathways were involved in the regulation of heat tolerance. Next, to investigate whether SL signaling pathways were involved in *AtMYBS1*-regulated heat tolerance, we constructed *atmybs1d14* double mutants and evaluated their tolerance to heat stress. Heat tolerance of the *atmybs1d14* double mutants was significantly lower than that of *atmybs1* mutants and similar to that of *d14* mutants (Figure 4C and 4F). $GR24^{4DO}$ application did not reverse the heat-hypersensitive phenotypes of *atmybs1d14* mutants (Figure 4C and 4F). In accordance with their different heat-response behaviors, *max1* and *atmybs1max1* double mutants showed upregulated expression of the heat-response genes *HSF3*, *HSP70*, and *HSP90* (Supplemental Figure 12) under $GR24^{4DO}$ treatment, whereas *atmybs1d14* did not show a significant difference (Supplemental Figure 12). In summary, we concluded that the SL signaling pathway was necessary for *AtMYBS1*–*MAX1*-mediated regulation of heat tolerance *in vivo*.

DISCUSSION

SLs are a new class of phytohormones involved in numerous plant physiological processes (Mashiguchi et al., 2021). Impairment of the SL pathway can cause hypersensitivity to several stresses, including drought, salt, and seed thermoinhibition (Mostofa et al., 2018). SL biosynthesis requires D27/AtD27, CCD7 (D17/MAX3/RMS5/DAD3), CCD8 (D10/MAX4/RMS1/DAD1), and CYP711As (e.g., A1(MAX1)/A2/A3) in a sequential manner (Al-Babili and Bouwmeester, 2015). Among SL biosynthesis enzymes, the CYP711A family, to which MAX1 belongs, plays an essential role in the biosynthesis of both canonical and noncanonical SLs (Mashiguchi et al., 2021). In this study, we revealed that AtMYBS1 functions as a negative regulator of heat tolerance by directly repressing *MAX1* expression. Both SL biosynthesis and signaling pathways are required for the regulation of heat tolerance by AtMYBS1. Our results thus provide new information related to SL.

(E) Statistical analysis of survival rates for the plants in **(B)**. After heat treatment, dead plants were counted and statistically analyzed. Plants whose shoot apices turned white were considered dead. Three independent biological replicates were performed ($n > 50$ for each replicate). Data are means ± SD; different letters on error bars indicate significant differences at $P < 0.05$, Tukey's *t*-test.

(F) Statistical analysis of survival rates for the plants in **(F)**. After heat treatment, dead plants were counted and statistically analyzed. Plants whose shoot apices turned white were considered dead. Three independent biological replicates were performed ($n > 50$ for each replicate). Data are means ± SD; different letters on error bars indicate significant differences at $P < 0.05$, Tukey's *t*-test.

The MYB binding site in the *MAX1* promoter is responsible for direct repression of *MAX1* by *AtMYBS1* in regulation of heat tolerance

We first found that *AtMYBS1* expression was downregulated by heat treatment (Figure 1A and Supplemental Figure 1A), and we then confirmed that *AtMYBS1* was a negative regulator of plant heat tolerance (Figure 1B). Phenotypic similarities between SL-deficient/insensitive mutants and *AtMYBS1*-OE plants prompted us to investigate whether the SL pathway might be regulated by *AtMYBS1 in vivo* (Brewer et al., 2009; Al-Babili and Bouwmeester, 2015). Our results showed that AtMYBS1 negatively regulates expression of the SL biosynthesis gene *MAX1* (Figure 2A). To investigate whether regulation of *MAX1* by AtMYBS1 takes part in the regulation of plant heat tolerance, we first examined the role of *MAX1* in the regulation of heat tolerance. The *MAX1* expression pattern and transgenic studies showed that *MAX1* played a positive role in regulating plant heat tolerance (Figure 2B and 2C). To investigate whether *AtMYBS1* regulates heat tolerance through *MAX1*, we evaluated the heat tolerance of *35S*:*MAX1*/*35S*:*AtMYBS1* plants and *atmybs1max1* double mutants. The results confirmed that *AtMYBS1*-regulated heat tolerance occurred through negative regulation of *MAX1* (Figure 2C).

To determine whether AtMYBS1 can directly regulate *MAX1*, we investigated their interactions by yeast one-hybrid assays *in vitro* and ChIP–qPCR *in vivo* (Figure 3A and 3B; Supplemental Figure 5). The results confirmed the direct interaction between AtMYBS1 and the *MAX1* promoter through the MYB binding site. We confirmed the transcriptional repression of *MAX1* by AtMYBS1 and the role of the MYB binding site in this repression using LUC reporter gene assays in *N. benthamiana* leaves (Figure 3C and Supplemental Figure 7A). We then analyzed the role of the MYB binding site in *AtMYBS1*–*MAX1*-regulated heat tolerance in two steps. In the first step, we investigated whether the MYB binding site was involved in regulation of heat tolerance by analyzing two types of transgenic plants, *pMAX1*:*gMAX1*/*max1* and *pMAX1m*:*gMAX1*/*max1*, in which the native promoter and a promoter with a mutated MYB binding site were used to drive *MAX1* expression in the *max1* mutant background (Supplemental Figure 7B). The results showed that mutation of the MYB binding site interfered with *MAX1* repression, confirming the necessary role of the MYB binding site in regulating plant heat tolerance (Figure 3D and Supplemental Figure 8). In the second step, we confirmed the function of the MYB binding site in *AtMYBS1*-regulated heat tolerance. We generated *35S*:*AtMYBS1max1* plants, in which *AtMYBS1* was overexpressed in the *max1* background. We then transformed *pMAX1*:*gMAX1* and *pMAX1m*:*gMAX1* vectors into *35S*:*AtMYBS1max1* plants (*pMAX1*:*gMAX1*/*35S*:*AtMYBS1max1* and *pMAX1m*:*gMAX1*/*35S*:*AtMYBS1max1*) and evaluated *MAX1* expression and heat tolerance in the transgenic plants. The results showed that mutation of the MYB binding site eliminated *AtMYBS1*-mediated repression of *MAX1*, confirming the necessary role of the MYB binding site in *AtMYBS1*-regulated heat tolerance (Figure 3E and Supplemental Figure 8).

SL biosynthesis and signaling pathways are required for *AtMYBS1*-regulated heat tolerance

Recent studies have shown that MAX1 is a key enzyme in the SL biosynthesis pathway (Al-Babili and Bouwmeester, 2015). Loss of function of *MAX1* leads to impairment of SL biosynthesis and further downstream signaling (Mashiguchi et al., 2021). However, loss of function of an enzyme causes not only a reduction in products but also an accumulation of substrates. Substrate accumulation may also have a large effect on plant development and stress responses. The substrate CL accumulated approximately 700-fold in *max1 mutants* compared with the control (Al-Babili and Bouwmeester, 2015). Despite having no SL activity, CL has been reported to affect the elongation of plant hypocotyls, indicating that it may have other functions in plants (Scaffidi et al., 2013; Al-Babili and Bouwmeester, 2015). Moreover, the cytochrome P450 enzymes to which MAX1 belongs have been shown to participate in various metabolic processes (Shang and Huang, 2020). Thus, the possibility that MAX1 might be involved in other metabolic pathways in addition to SL biosynthesis cannot be excluded. Based on the above considerations, although *AtMYBS1* functions by regulating *MAX1* expression, we could not assume that *AtMYBS1*-regulated heat tolerance must be realized through SL biosynthesis and signaling pathways. We therefore performed further studies to investigate this issue.

In addition to analyzing *MAX1*, we also analyzed the roles of two other SL biosynthesis genes, *MAX3* and *MAX4*, in the regulation of plant heat tolerance. The *MAX3* gene encodes carotenoid cleavage dioxygenase 7 (CCD7), which catalyzes the stereospecific cleavage of 9-*cis*-β-carotene to produce 9-*cis*-β-apo-10′-carotenal and β-ionone. *MAX4* encodes CCD8, which catalyzes the conversion of 9-*cis*-β-apo-10′-carotenal to CL, the substrate of MAX1 (Omoarelojie et al., 2019). We first evaluated the heat tolerance of *max3*, *max4*, *35S*:*MAX3*, and *35S*:*MAX4* plants and found that *MAX3* and *MAX4* had roles in regulating plant heat tolerance similar to that of *MAX1* (Figure 4A). We overexpressed *MAX1* in the *max3* or *max4* background (*35S*:*MAX1*/*max3* or *35S*:*MAX1*/*max3*) and found that deficiency in MAX3 and MAX4 products interferes with MAX1 function in regulating heat tolerance (Figure 4A). We also treated *max1*, *max3*, *max4*, *35S*:*MAX1*/*max3*, *35S*:*MAX1*/*max4*, *AtMYBS1*-OE, and *atmybs1max1* plants with the SL analog $GR24^{4DO}$ and found that $GR24^{4DO}$ reversed the heat hypersensitivity of all these plants (Figure 4B and 4C), confirming the role of SL biosynthesis in regulating plant heat tolerance. These results, combined with those from *35S*:*MAX1*/*35S*:*AtMYBS1* and *atmybs1max1* plants (Figure 2C), led us to conclude that the SL biosynthesis pathway was required for *AtMYBS1*-regulated heat tolerance, although SL contents could not be measured *in vivo* in *Arabidopsis* because of technical limitations.

We found that the expression of *MAX3* and *MAX4* decreased slightly in response to heat stress (Supplemental Figure 10), in contrast to the expression pattern of *MAX1* (Figure 2B). However, the degree of change in *MAX3* and *MAX4* expression was much smaller than that in *MAX1* (Figure 2B and Supplemental Figure 10). We speculated that the downregulation of *MAX3* and *MAX4* might be indirect and due to negative feedback regulation by the upregulation of *MAX1* in response to heat stress. The expression of *MAX3* and *MAX4* was significantly enhanced in *AtMYBS1*-OE plants but slightly decreased in *atmybs1* mutants (Figure 2A). Increased expression levels of *MAX3*/*CCD7* and *MAX4*/*CCD8* were previously reported in SL-deficient and SL-insensitive mutants of several plant species, such as *Arabidopsis*,

pea, petunia, and rice (Foo et al., 2005; Snowden et al., 2005; Johnson et al., 2006; Umehara et al., 2008; Arite et al., 2009; Drummond et al., 2009; Hayward et al., 2009; Mashiguchi et al., 2009). Upregulation of *CCD7* and *CCD8* could be reversibly counteracted by exogenous application of GR24, a synthetic SL analog, in wild-type and SL-deficient plants (Umehara et al., 2008; Mashiguchi et al., 2009). Levels of 4DO and SL biosynthetic intermediates such as CL and CLA were also markedly increased in SL-insensitive mutants of *Arabidopsis* or rice (Umehara et al., 2008; Arite et al., 2009; Abe et al., 2014; Seto et al., 2014). This evidence strongly supports the notion that SL biosynthesis is controlled by negative feedback regulation. Because SLs are involved in the regulation of various plant activities, their levels should be carefully modulated as part of a homeostatic steady state, which might explain the significance of the negative feedback mechanism of SL biosynthesis (Koltai and Beveridge, 2013). Among SL biosynthesis enzymes, the cytochrome P450 enzyme MAX1 and its homologs are essential and convert CL to CLA, which is further processed into diverse canonical and noncanonical SLs (Zhang et al., 2014; Yoneyama et al., 2018; Wakabayashi et al., 2019; Burger, 2021). Thus, the most efficient way to regulate SL biosynthesis might be through direct control of *MAX1* expression or MAX1 enzyme activity, which may have significance for plant adaptation to rapidly changing conditions. The actual molecular mechanisms that underlie different expression patterns of *MAX3*, *MAX4*, and *MAX1* in response to heat stress are interesting and need to be elucidated in future studies.

To investigate whether *AtMYBS1*–*MAX1*-regulated heat tolerance depends on the SL signaling pathway, we performed studies on the SL receptor gene *d14*, which encodes an α/β-hydrolase (Waters et al., 2017). We first wanted to determine the roles of *d14* in SL-regulated heat tolerance. Evaluation of heat tolerance in *d14*, *35S*:*MAX1d14*, and *atmybs1d14* plants with or without $GR24^{4DO}$ treatment revealed that *d14* was required for SL-mediated regulation of heat tolerance (Figure 4C and 4F). We next investigated the role of *d14* in *AtMYBS1*-regulated heat tolerance by evaluating the heat tolerance of *atmybs1d14* double mutants with or without $GR24^{4DO}$ application (Figure 4C and 4F). The results confirmed the necessary role of the SL signaling pathway in *AtMYBS1*–*MAX1*-regulated heat tolerance.

Molecular mechanisms underlying the regulation of heat and salt stress responses by *AtMYBS1*

Previous studies have shown that AtMYBS1/AtMYBL functions as a transcription factor involved in responses to salt stress by regulating the ABA and sugar pathways (Lu et al., 2002; Zhang et al., 2011; Chen et al., 2017). *AtMYBS1*-OE transgenic plants had an improved seed germination rate under salt-stress conditions (Zhang et al., 2011). However, when the survival rates of 14-day-old seedlings were evaluated, *AtMYBS1*-OE plants displayed salt-sensitive phenotypes, whereas the *atmybs1* mutant was resistant (Zhang et al., 2011). Accordingly, expression of the stress marker genes *RD29A* and *RD29B* was decreased in *AtMYBS1*-OE plants but increased in *atmybs1* mutants (Zhang et al., 2011). The different seed germination phenotypes and survival rates of 14-day-old seedlings under salt stress indicated that *AtMYBS1* might function developmentally in regulating stress sensitivity. The *atmybs1* mutants were hypersensitive to ABA, and the ABA biosynthesis genes *ABA1*, *NECD9*, and *AAO3* and the ABA signaling genes *ABI3*, *ABI4*, and *ABI5* were upregulated (Chen et al., 2017). These results indicated that *atmybs1* mutants might have an increased level of ABA *in vivo* (Chen et al., 2017). In addition, expression of HXK1, a glucose sensor, was increased in *atmybs1* mutants, indicating that *AtMYBS1* might negatively regulate the sugar pathway (Rolland et al., 2006; Chen et al., 2017). Previous studies have shown that glucose enhances the ABA pathway through the *HXK*-dependent sugar signaling pathway (Arenas-Huertero et al., 2000; Cheng et al., 2002). Therefore, enhancement of the ABA pathway in the *atmybs1* mutant might be due to increased expression of *HXK1*. In our studies, seedling survival rates, but not seed germination rates, were evaluated for their tolerance to heat stress. Our results were similar to those of previous studies in which overexpression of *AtMYBS1* resulted in hypersensitivity to stress and loss of function of *AtMYBS1* resulted in resistance, confirming the negative role of *AtMYBS1* in regulating plant stress responses (Zhang et al., 2011).

AtMYBS1 was downregulated by heat stress in our study, but it was induced by salt stress in previous work (Zhang et al., 2011). Salt stress may cause osmotic stress and ionic toxicity (Munns and Tester, 2008). When osmotic stress occurs, plants close the stomata to reduce transpirational water loss (Munemasa et al., 2015). By contrast, plants open the stomata when heat stress occurs and benefit from increased evaporative cooling (Urban et al., 2017). Therefore, different molecular mechanisms may underlie the responses to these two stresses. This may explain why *AtMYBS1* exhibited different expression patterns in response to salt and heat stresses, a possibility that will require further investigation in the future.

ABA is a stress hormone that plays an important role in regulating plant responses to different stresses (Bharath et al., 2021). The SL pathway was also found to interact with the ABA pathway. SL has been reported to induce the expression of *HB40*, which directly activates transcription of the ABA biosynthesis gene *AtNCED3* (Gonzalez-Grandio et al., 2017; Wang et al., 2020). In our studies, we found that *AtMYBS1* negatively regulates the SL pathway. Thus, the ABA pathway may have been influenced by *AtMYBS1*, although this will need to be confirmed in future studies. As a transcription factor, AtMYBS1 may have thousands of target genes. For example, the *AtMYBS1* homolog in rice was shown to bind to the promoter of α-amylase *in vitro* (Lu et al., 2002). Comprehensive analysis of AtMYBS1 target genes by ChIP sequencing may be helpful for elucidating the regulatory network of AtMYBS1 in response to different stresses. The roles of *AtMYBS1* in regulating heat tolerance and branch number have not been reported previously, and our results provide new insights into the function of *AtMYBS1*.

On the basis of our results, we propose a functional model for the regulation of heat tolerance by *AtMYBS1* in *Arabidopsis* (Figure 5). Expression of *AtMYBS1* is downregulated by heat stress, releasing the direct repression of *MAX1* by AtMYBS1 through the MYB binding site in the *MAX1* promoter. Increased *MAX1* expression activates heat-resistance mechanisms through the SL signaling pathway and confers heat resistance to plants.

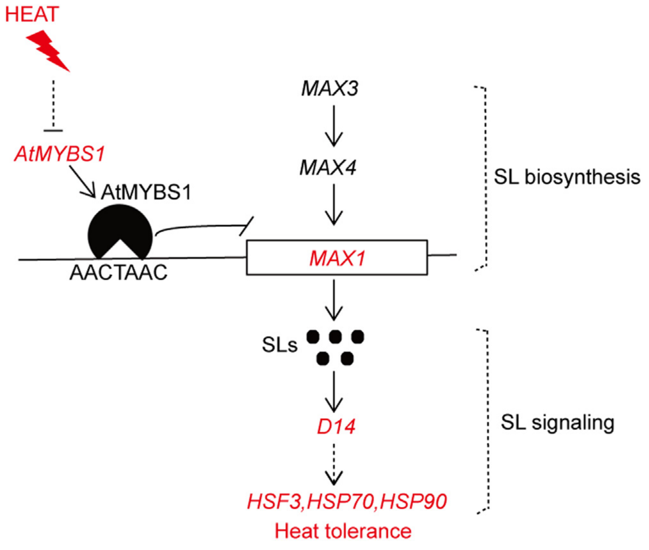

Figure 5. Working model for the regulation of heat tolerance by *AtMYBS1–MAX1* in *Arabidopsis.*

AtMYBS1 directly represses *MAX1* expression through the MYB binding site in the *MAX1* promoter. Heat stress represses *AtMYBS1* expression, thereby releasing the repression of *MAX1* by AtMYBS1. Increased expression of *MAX1* activates the SL pathway and thereafter heat-resistance mechanisms, such as enhanced expression of heat-responsive genes (*HSF3*, *HSP70*, *HSP9*), to confer heat resistance to plants.

Our studies thus add to the current understanding of the SL pathway in plant development and stress responses.

METHODS

Plant materials

All plants used in this study were in the *Arabidopsis thaliana* Columbia genetic background. The *atmybs1-1* (CS843799) and *atmybs1-2* (CS806410) mutants were ordered from the SALK collections (https://www.arabidopsis.org/). The *max1* mutant line *max1-1* and *max4* mutant line *max4-1* were provided by Professor Qingyun Bu, Chinese Academy of Sciences (Stirnberg et al., 2002; Sorefan et al., 2003). The *max3* mutant line *max3-9* and *d14* mutant line *d14-1* were obtained from Professor Jiayang Li, Chinese Academy of Sciences (Wang et al., 2015). Primers for genotyping homozygous *atmybs1-1* and *atmybs1-2* mutants are listed in Supplemental Table 1.

The *35S*:*AtMYBS1* and *35S*:*AtMYBS1-6×HA* plants were obtained by transforming the *35S*:*AtMYBS1* (*pJL12* vector) and *35S*:*AtMYBS1* (*pJL12-6×HA*) constructs into Col-0 plants using the floral dip method (-Clough and Bent, 1998). To screen homozygous transgenic plants, T_1-positive plants were selected by spraying 20 mg/l glufosinate ammonium on all sown seeds (three times with a 2-day interval). T_2 plants were plated onto half-strength Murashige and Skoog (MS) medium containing 10 mg/l glufosinate ammonium, and lines with survival ratios matching 3:1 were reserved and propagated. The T_3 seeds were harvested and selected in half-strength MS medium containing 10 mg/l glufosinate ammonium, and lines with a 100% survival rate were considered homozygous. To generate *atmybs1max1* and *atmybs1d14* double mutants, *atmbs1-1* (paternal) was crossed with *max1-1* or *d14-1* mutants (maternal). The obtained F_1 generation plants continued to undergo self-pollination and yielded F_2 generation seeds. To screen *atmybs1max1* and *atmybs1d14* double homozygous mutants, F_3 generation plants with excessive branching (*max1* or *d14* background) were selected and genotyped for the *atmybs1* background. *35S*:*MAX1*, *35S*:*MAX1*/*35S*:*AtMYBS1*, *35S*:*MAX1d14*, *35S*:*MAX1max3*, and *35S*:*MAX1max4* plants were generated by transforming the *35S*:*MAX1* (*pMDC85* vector) construct into Col-0, *35S*:*AtMYBS1*, *d14*, *max3*, and *max4* mutants, respectively. To screen homozygous transgenic plants, T_2 plants with a survival ratio of 3:1 were propagated, and T_3 seeds with a 100% survival rate on half-strength MS medium containing 30 mg/l hygromycin were used as homozygous transgenic lines. The *35S*:*MAX3* and *35S*:*MAX4* plants were obtained by transforming *35S*:*MAX3* (*pJL12* vector) and *35S*:*MAX4* (*pJL12* vector) constructs into Col-0 plants. To screen homozygous transgenic plants, T_2 plants with a survival ratio of 3:1 were propagated, and T3 seeds with a 100% survival rate on half-strength MS medium containing 10 mg/l glufosinate ammonium were used as homozygous transgenic lines. *pMAX1*:*gMAX1*/*max1* and *pMAX1m*:*gMAX1*/*max1* plants were obtained by transforming *pMAX1*:*gMAX1* (*p1300* vector) or *pMAX1m*:*gMAX1* (*p1300* vector) constructs into *max1* mutants. To screen homozygous transgenic plants, T_2 plants with a survival ratio of 3:1 were propagated, and T_3 seeds with a 100% survival rate on half-strength MS medium containing 30 mg/l hygromycin were used as homozygous transgenic lines. *pMAX1*:*gMAX1*/*35S*:*AtMYBS1max1* and *pMAX1m*:*gMAX1*/*35S*:*AtMYBS1max1* plants were obtained by transforming *pMAX1*:*gMAX1* (*p1300* vector) or *MAX1m*:*gMAX1* (*p1300* vector) constructs into *35S*:*AtMYBS1max1* plants. To screen homozygous transgenic plants, T_2 plants with a survival ratio of 3:1 were propagated, and T_3 seeds with a 100% survival rate on half-strength MS medium containing 30 mg/l hygromycin were used as homozygous transgenic lines.

All plants were grown in a greenhouse (23°C, 75% humidity, 60–80 μE m^{-2} s^{-1} light intensity, 16 h light/8 h dark cycle).

Plasmid construction and plant transformation

All constructs in this study were created using the Vazyme one-step cloning kit (Vazyme, China, cat. #C115-01). Primers for plasmid construction are listed in Supplemental Table 1. Plant transformation was performed by the floral dip method (Clough and Bent, 1998). In brief, to generate the *pMAX1m*:*LUC* plasmid, primers pMAX1m-pGreenII0800-F1 and pMAX1m-pGreenII0800-R1 were used to amplify the first fragment of the *MAX1* promoter (Supplemental Table 1), and primers pMAX1m-pGreenII0800-F2 and pMAX1m-pGreenII0800-R2 were used to amplify the second fragment of the *MAX1* promoter (Supplemental Table 1). The two fragments were recovered and mixed as templates to amplify the mutated *MAX1* promoter using primers pMAX1m-pGreenII0800-F1 and pMAX1m-pGreenII0800-R2. The amplified mutated *MAX1* promoter was then integrated into the *pGreenII0800* vector. To generate the *pMAX1m*:*gMAX1* plasmid, primers pMAX1m-gMAX1-F1 and pMAX1m-gMAX1-R1 were used to amplify the first part of the *MAX1* genomic sequence, and primers pMAX1m-gMAX1-F2 and pMAX1m-gMAX1-R2 were used to amplify the second part of the *MAX1* genomic sequence (Supplemental Table 1). These two fragments were then recovered and mixed to amplify the mutated *MAX1* genomic fragment. Finally, the mutated *MAX1* genomic fragment was integrated into the pCAMBIA1300 vector.

Heat treatment, GR24^{4DO} application, branching phenotype observation, and statistical analysis

To examine gene expression patterns, 12-day-old seedlings grown at 23°C in half-strength MS medium were exposed to 40°C in a climate chamber (40°C, 60% humidity, 80–100 μE m^{-2} s^{-1}, 16 h light/8 h dark cycle) for the indicated times, then used for qRT−PCR or GUS staining assays.

For heat-tolerance analysis, heat treatments were performed as described in a previous study, with minor modifications (Hong and Vierling, 2000). In brief, seeds of different genotypes were sterilized, sown onto half-strength MS medium, and grown in the greenhouse (23°C, 75% humidity, 60–80 μE m^{-2} s^{-1}, 16 h light/8 h dark cycle). Seedlings with two true leaves (14 days) were exposed to 40°C for 6 h in a

climate chamber (40°C, 60% humidity, 80–100 μE m^{-2} s^{-1}, 16 h light/8 h dark cycle), followed by 2 h of recovery at 23°C in the greenhouse. For survival analysis, plants whose shoot apices had turned white were considered to be dead. Plant death rates were calculated and statistically analyzed. The original whole-dish photos for Figures 1B, 2C, 3D, 3E, and 4A–4C are provided in Supplemental Figure 13.

For SL treatment, $GR24^{4DO}$ was purchased from StrigoLab (Italy, cat. #EN4) and dissolved in isopropanol to prepare 0.1 mM solutions. Fourteen-day-old seedlings of different genotypes grown on half-strength MS medium in the greenhouse (23°C, 75% humidity, 60–80 μE m^{-2} s^{-1}, 16 h light/8 h dark cycle) were sprayed with 10 μM $GR24^{4DO}$. The treated plants continued to grow in the greenhouse for 8 h (overnight). Subsequently, the $GR24^{4DO}$-treated plants were subjected to heat-stress treatment.

For branching observation and statistical analysis, as described in a previous report (Brewer et al., 2016), buds with lengths over 5 mm were defined as newly developed branches. Seeds were sown and grown in the greenhouse (23°C, 75% humidity, 60–80 μE m^{-2} s^{-1}, 16 h light/8 h dark cycle). The different lines did not show large differences in flowering time. After all plants had bolted and flowered (approximately 6 weeks, primary branch over 10 cm and self-fertilized), samples (n > 10) were collected, and their primary branches were counted and statistically analyzed. All statistical analyses were performed using Tukey's t-test.

RNA extraction and qRT−PCR analysis

Total RNA was extracted from 2-week-old seedlings with or without heat treatment using an RNAprep Pure Plant Kit (Tiangen, China, cat. #DP441). cDNA was synthesized according to the manufacturer's instructions (Clontech, Japan, cat. #6110A), and qRT−PCR was performed on a 484 ABI 7500 real-time PCR system using the SYBR Green Mix Kit (Bio-Rad, Hercules, CA, USA). *ACTIN7* (At5g09180) was used as an internal control. Primers for qRT−PCR are listed in Supplemental Table 1.

GUS staining and activity assay

For GUS staining, a 2613-bp genomic fragment upstream of ATG at the *AtMYBS1* locus was amplified, integrated into the pKGWFS7 vector, and transformed into Col-0. GUS staining was performed as described previously (Li et al., 2020).

For the GUS activity assay, GUS activity was quantified using 4-methylumbelliferyl β-D-glucuronide (4-MUG) as the substrate. First, we collected 500 mg of seedling tissue for each sample and ground it into fine powder in liquid nitrogen; we then added 150 μl of GUS extraction buffer (10 mM EDTA [pH 8.0], 0.1% SDS, 50 mM sodium phosphate [pH 7.0], 0.1% Triton X-100, and 10 mM β-mercaptoethanol, with 25 mg/ml phenylmethylsulfonyl fluoride added before use), centrifuged the samples at 15 000 rpm for 10 min, transferred the supernatants to microtubes, and kept them on ice. Second, we prepared a reaction mix (GUS extraction buffer with 1 mM 4-MUG) for each sample, added 1 ml of reaction buffer to microcentrifuge tubes, and prewarmed the tubes at 37°C; 10 μl of the supernatant was then added to the reaction tubes at 30-s intervals and incubated for 10 min, and 100 μl of reaction solution was added to vials containing 1 M sodium carbonate to stop the reaction. Third, we diluted 4-methyl umbelliferone (4-MU) stock solutions to 100 nM, 200 nM, and 400 nM in order to plot a standard curve at an excitation wavelength of 365 nm, emission wavelength of 455 nm, and filter wavelength of 430 nm. We measured the fluorescence of each sample and calculated the amount of 4-MU according to the standard curve. Finally, we quantified the total protein concentration of each sample and determined the GUS activity.

Yeast one-hybrid assay

A yeast one-hybrid assay was performed according to the manufacturer's instructions (Clontech, Japan, cat. #630491, #630466, #630499). In brief, we first amplified the bait sequences (segments in the *MAX1* promoter) and incorporated them into the pAbAi vector. We then used the *Bst*BI restriction enzyme to linearize the constructed vectors and transformed them into the yeast strain Y1H Gold. The bait sequences were integrated into the yeast genome via recombination. After selection on synthetic defined (SD) medium without uracil (SD-Ura), we picked healthy colonies for PCR validation. To avoid false-positive errors, the selected yeast colonies were screened on SD-Ura medium supplemented with an appropriate concentration of aureobasidin A (AbA), in the presence of which yeast cells do not grow. Next, the prey vector pGADT7-AtMYBS1 was generated and transformed into the Y1H Gold strains containing the pAbAi-bait vectors. We used SD-Leu selective medium to select positive colonies and subsequently validated them by PCR amplification. Finally, Y1H Gold yeast strains harboring both the pGADT7-AtMYBS1 and pAbAi-bait vectors were plated on SD-Ura-Leu medium containing 50 ng/ml AbA to examine direct interactions between AtMYBS1 and *MAX1* promoters.

Western blotting and ChIP−qPCR

Western blotting and ChIP−qPCR were performed as described previously (An et al., 2017). In brief, for the western blot assay, total proteins were extracted from Col-0 and *35S:AtMYBS1-6XHA#7*, separated in a 10% polyacrylamide gel, and transferred onto a polyvinylidene fluoride membrane. After blocking, the membrane was sequentially incubated with primary antibody (anti-HA, Abcam, UK, #ab18181) and secondary antibody (mouse HRP, Abcam, #ab131368) at room temperature for 2 h. The chemiluminescent signal was detected using an Enhanced Chemifluorescent HRP Substrate Kit (Thermo Fisher, USA, cat. #15159).

For the ChIP−qPCR assay, 2 g of 2-week-old seedlings of Col-0 and *35S:AtMYBS1-6XHA#7* were ground to fine powder, crosslinked in 1% formaldehyde for 30 min, and neutralized in 0.125 M glycine. The samples were subjected to cell lysis and shearing by sonication (to reduce the DNA fragments to approximately 500 bp). Prior to co-immunoprecipitation, the samples were cleared with Protein A salmon sperm–coupled agarose (Sigma−Aldrich, USA, cat. #16-157). The chromatin samples were then immunoprecipitated overnight at 4°C with HA antibodies (Abcam, #ab18181). Next, the immunoprecipitated chromatin complexes were incubated with protein A salmon sperm–coupled agarose (Sigma−Aldrich, #16-157) and subjected to a series of washing procedures with low salt concentration buffer, high salt concentration buffer, LiCl buffer, and TE buffer. Finally, the immunoprecipitated chromatin was eluted with elution buffer (1% SDS, 0.1 M $NaHCO_3$). Protein–DNA crosslinking was reversed by incubating the immunoprecipitated complexes at 65°C overnight. DNA was recovered using a QIAquick PCR Purification Kit (Qiagen, USA, cat. #28106) and analyzed by real-time qPCR. *ACTIN7* (At5g09810) was used as a nonspecific target gene locus. Primers for qPCR are listed in Supplemental Table 1.

Transcriptional activity assay

Luciferase reporter assays were performed to investigate the transcriptional activity of AtMYBS1. First, the *AtMYBS1* coding sequence was cloned and inserted into the *PJL12-GFP* vector to generate the *35S:AtMYBS1-GFP* construct. Second, the *MAX1* promoter (2050 bp upstream of ATG) was cloned and inserted into *pGreenII0800* to generate the *pMAX1:LUC* construct. The mutated MYB binding site (AACTCCG) in the *MAX1* promoter was also cloned and inserted into *pGreenII0800* to generate the *pMAX1m:LUC* construct. The empty vector *PJL12-GFP* (35S:GFP) and *pGreenII0800* were used as controls. All the above vectors were transformed into *Agrobacterium tumefaciens* (strain GV310). Before infiltration, the GV3101 strains were harvested and resuspended in 2-(*N-morpholino)ethanesulfonic acid* (MES) buffer (10 mM $MgCl_2$, 10 mM MES, 20 μM acetosyringone [pH 5.7]) and kept in the dark at room temperature for at least 2 h. For different infiltration sets, equal volumes of strains were mixed and injected into *N. benthamiana* leaves. The infiltrated leaves were sprayed with 10 mM lucoferin (Promega) at 48 h post infiltration and

kept in the dark for 5 min before luminescence was recorded using the Nightshade LB 985 *in vivo* Plant Imaging System (Berthold Technologies, Bad Wildbad, Germany). Three independent biological replicates were examined for each set of assays, and each replicate consisted of four leaves from four separate plants. The LUC reporter assays were repeated three times.

SUPPLEMENTAL INFORMATION

Supplemental information is available at *Plant Communications Online*.

FUNDING

This work was supported by Henan Agricultural University (30500689).

AUTHOR CONTRIBUTIONS

Y.J. conceived the study. X.L. performed most of the experiments. J.L., X.Z., Y.D., Y.L., S.C., E.X., and X.Z. helped with vector construction, transformation, and data analysis. Y.J., X.L., E.X., and X.Z. analyzed the data and wrote and revised the article.

ACKNOWLEDGMENTS

We thank Professor Jiayang Li, Chinese Academy of Sciences, for sharing seeds of the *max3* and *d14* mutants and Professor Qingyun Bu, Chinese Academy of Sciences, for sharing seeds of the *max1* and *max4* mutants. We thank Professors Jiayang Li and Bing Wang, Chinese Academy of Sciences, Professor Zefu Lu, Chinese Academy of Agricultural Sciences, and Professor Jing Wang, Sichuan Agricultural University, for their advice on the manuscript. We thank Professor Shengyi Liu, Yizhou He, and Huqman Bin Safdar for providing technical support for this study. No conflict of interest is declared.

Received: April 5, 2023
Revised: July 20, 2023
Accepted: August 18, 2023
Published: August 22, 2023

REFERENCES

Abe, S., Sado, A., Tanaka, K., Kisugi, T., Asami, K., Ota, S., Kim, H.I., Yoneyama, K., Xie, X., Ohnishi, T., et al. (2014). Carlactone is converted to carlactonoic acid by MAX1 in *Arabidopsis* and its methyl ester can directly interact with AtD14 in vitro. Proc. Natl. Acad. Sci. USA **111**:18084–18089. https://doi.org/10.1073/pnas.1410801111.

Al-Babili, S., and Bouwmeester, H.J. (2015). Strigolactones, a novel carotenoid-derived plant hormone. Annu. Rev. Plant Biol. **66**:161–186. https://doi.org/10.1146/annurev-arplant-043014-114759.

Alder, A., Jamil, M., Marzorati, M., Bruno, M., Vermathen, M., Bigler, P., Ghisla, S., Bouwmeester, H., Beyer, P., and Al-Babili, S. (2012). The path from beta-carotene to carlactone, a strigolactone-like plant hormone. Science **335**:1348–1351. https://doi.org/10.1126/science.1218094.

An, C., Li, L., Zhai, Q., You, Y., Deng, L., Wu, F., Chen, R., Jiang, H., Wang, H., Chen, Q., and Li, C. (2017). Mediator subunit MED25 links the jasmonate receptor to transcriptionally active chromatin. Proc. Natl. Acad. Sci. USA **114**:E8930–E8939. https://doi.org/10.1073/pnas.1710885114.

Arenas-Huertero, F., Arroyo, A., Zhou, L., Sheen, J., and León, P. (2000). Analysis of *Arabidopsis* glucose insensitive mutants, gin5 and gin6, reveals a central role of the plant hormone ABA in the regulation of plant vegetative development by sugar. Genes Dev. **14**:2085–2096.

Arite, T., Umehara, M., Ishikawa, S., Hanada, A., Maekawa, M., Yamaguchi, S., and Kyozuka, J. (2009). d14, a strigolactone-insensitive mutant of rice, shows an accelerated outgrowth of tillers. Plant Cell Physiol. **50**:1416–1424. https://doi.org/10.1093/pcp/pcp091.

Bennett, T., Sieberer, T., Willett, B., Booker, J., Luschnig, C., and Leyser, O. (2006). The *Arabidopsis* MAX pathway controls shoot branching by regulating auxin transport. Curr. Biol. **16**:553–563. https://doi.org/10.1016/j.cub.2006.01.058.

Bharath, P., Gahir, S., and Raghavendra, A.S. (2021). Abscisic Acid-Induced Stomatal Closure: An Important Component of Plant Defense Against Abiotic and Biotic Stress. Front. Plant Sci. **12**, 615114. https://doi.org/10.3389/fpls.2021.615114.

Booker, J., Sieberer, T., Wright, W., Williamson, L., Willett, B., Stirnberg, P., Turnbull, C., Srinivasan, M., Goddard, P., and Leyser, O. (2005). MAX1 encodes a cytochrome P450 family member that acts downstream of MAX3/4 to produce a carotenoid-derived branch-inhibiting hormone. Dev. Cell **8**:443–449. https://doi.org/10.1016/j.devcel.2005.01.009.

Boyer, F.D., de Saint Germain, A., Pillot, J.P., Pouvreau, J.B., Chen, V.X., Ramos, S., Stévenin, A., Simier, P., Delavault, P., Beau, J.M., and Rameau, C. (2012). Structure-activity relationship studies of strigolactone-related molecules for branching inhibition in garden pea: molecule design for shoot branching. Plant Physiol. **159**:1524–1544. https://doi.org/10.1104/pp.112.195826.

Brewer, P.B., Dun, E.A., Ferguson, B.J., Rameau, C., and Beveridge, C.A. (2009). Strigolactone acts downstream of auxin to regulate bud outgrowth in pea and *Arabidopsis*. Plant Physiol. **150**:482–493. https://doi.org/10.1104/pp.108.134783.

Brewer, P.B., Yoneyama, K., Filardo, F., Meyers, E., Scaffidi, A., Frickey, T., Akiyama, K., Seto, Y., Dun, E.A., Cremer, J.E., et al. (2016). LATERAL BRANCHING OXIDOREDUCTASE acts in the final stages of strigolactone biosynthesis in *Arabidopsis*. Proc. Natl. Acad. Sci. USA **113**:6301–6306. https://doi.org/10.1073/pnas.1601729113.

Bürger, M. (2021). Insights into the evolution of strigolactone signaling. Plant Cell **33**:3389–3390. https://doi.org/10.1093/plcell/koab216.

Bürger, M., and Chory, J. (2020). The Many Models of Strigolactone Signaling. Trends Plant Sci. **25**:395–405. https://doi.org/10.1016/j.tplants.2019.12.009.

Chen, Y.S., Chao, Y.C., Tseng, T.W., Huang, C.K., Lo, P.C., and Lu, C.A. (2017). Two MYB-related transcription factors play opposite roles in sugar signaling in *Arabidopsis*. Plant Mol. Biol. **93**:299–311. https://doi.org/10.1007/s11103-016-0562-8.

Cheng, W.H., Endo, A., Zhou, L., Penney, J., Chen, H.C., Arroyo, A., Leon, P., Nambara, E., Asami, T., Seo, M., et al. (2002). A unique short-chain dehydrogenase/reductase in *Arabidopsis* glucose signaling and abscisic acid biosynthesis and functions. Plant Cell **14**:2723–2743. https://doi.org/10.1105/tpc.006494.

Clough, S.J., and Bent, A.F. (1998). Floral dip: a simplified method for Agrobacterium-mediated transformation of *Arabidopsis thaliana*. Plant J. **16**:735–743. https://doi.org/10.1046/j.1365-313x.1998.00343.x.

Decker, E.L., Alder, A., Hunn, S., Ferguson, J., Lehtonen, M.T., Scheler, B., Kerres, K.L., Wiedemann, G., Safavi-Rizi, V., Nordzieke, S., et al. (2017). Strigolactone biosynthesis is evolutionarily conserved, regulated by phosphate starvation and contributes to resistance against phytopathogenic fungi in a moss, Physcomitrella patens. New Phytol. **216**:455–468. https://doi.org/10.1111/nph.14506.

Drummond, R.S.M., Martínez-Sánchez, N.M., Janssen, B.J., Templeton, K.R., Simons, J.L., Quinn, B.D., Karunairetnam, S., and Snowden, K.C. (2009). Petunia hybrida CAROTENOID CLEAVAGE DIOXYGENASE7 is involved in the production of negative and positive branching signals in petunia. Plant Physiol. **151**:1867–1877. https://doi.org/10.1104/pp.109.146720.

Dubos, C., Stracke, R., Grotewold, E., Weisshaar, B., Martin, C., and Lepiniec, L. (2010). MYB transcription factors in *Arabidopsis*. Trends Plant Sci. **15**:573–581. https://doi.org/10.1016/j.tplants.2010.06.005.

Foo, E., Bullier, E., Goussot, M., Foucher, F., Rameau, C., and Beveridge, C.A. (2005). The branching gene RAMOSUS1 mediates interactions among two novel signals and auxin in pea. Plant Cell **17**:464–474. https://doi.org/10.1105/tpc.104.026716.

González-Grandío, E., Pajoro, A., Franco-Zorrilla, J.M., Tarancón, C., Immink, R.G.H., and Cubas, P. (2017). Abscisic acid signaling is controlled by a BRANCHED1/HD-ZIP I cascade in *Arabidopsis* axillary buds. Proc. Natl. Acad. Sci. USA **114**:E245–E254. https://doi.org/10.1073/pnas.1613199114.

Ha, C.V., Leyva-González, M.A., Osakabe, Y., Tran, U.T., Nishiyama, R., Watanabe, Y., Tanaka, M., Seki, M., Yamaguchi, S., Dong, N.V., et al. (2014). Positive regulatory role of strigolactone in plant responses to drought and salt stress. Proc. Natl. Acad. Sci. USA **111**:851–856. https://doi.org/10.1073/pnas.1322135111.

Hayward, A., Stirnberg, P., Beveridge, C., and Leyser, O. (2009). Interactions between auxin and strigolactone in shoot branching control. Plant Physiol. **151**:400–412. https://doi.org/10.1104/pp.109.137646.

Hong, S.W., and Vierling, E. (2000). Mutants of *Arabidopsis thaliana* defective in the acquisition of tolerance to high temperature stress. Proc. Natl. Acad. Sci. USA **97**:4392–4397. https://doi.org/10.1073/pnas.97.8.4392.

Johnson, X., Brcich, T., Dun, E.A., Goussot, M., Haurogné, K., Beveridge, C.A., and Rameau, C. (2006). Branching genes are conserved across species. Genes controlling a novel signal in pea are coregulated by other long-distance signals. Plant Physiol. **142**:1014–1026. https://doi.org/10.1104/pp.106.087676.

Koltai, H., and Beveridge, C. (2013). Strigolactones and the Coordinated Development of Shoot and Root (Springer). **Book Chapter**.

Lesk, C., Rowhani, P., and Ramankutty, N. (2016). Influence of extreme weather disasters on global crop production. Nature **529**:84–87. https://doi.org/10.1038/nature16467.

Li, Y., Xia, T., Gao, F., and Li, Y. (2020). Control of Plant Branching by the CUC2/CUC3-DA1-UBP15 Regulatory Module. Plant Cell **32**:1919–1932. https://doi.org/10.1105/tpc.20.00012.

Lobell, D.B., Schlenker, W., and Costa-Roberts, J. (2011). Climate trends and global crop production since 1980. Science **333**:616–620. https://doi.org/10.1126/science.1204531.

Lu, C.A., Ho, T.h.D., Ho, S.L., and Yu, S.M. (2002). Three novel MYB proteins with one DNA binding repeat mediate sugar and hormone regulation of alpha-amylase gene expression. Plant Cell **14**:1963–1980. https://doi.org/10.1105/tpc.001735.

Marzec, M., and Brewer, P. (2019). Binding or Hydrolysis? How Does the Strigolactone Receptor Work? Trends Plant Sci. **24**:571–574. https://doi.org/10.1016/j.tplants.2019.05.001.

Mashiguchi, K., Seto, Y., and Yamaguchi, S. (2021). Strigolactone biosynthesis, transport and perception. Plant J. **105**:335–350. https://doi.org/10.1111/tpj.15059.

Mashiguchi, K., Sasaki, E., Shimada, Y., Nagae, M., Ueno, K., Nakano, T., Yoneyama, K., Suzuki, Y., and Asami, T. (2009). Feedback-regulation of strigolactone biosynthetic genes and strigolactone-regulated genes in *Arabidopsis*. Biosci. Biotechnol. Biochem. **73**:2460–2465. https://doi.org/10.1271/bbb.90443.

Mashiguchi, K., Seto, Y., Onozuka, Y., Suzuki, S., Takemoto, K., Wang, Y., Dong, L., Asami, K., Noda, R., Kisugi, T., et al. (2022). A carlactonoic acid methyltransferase that contributes to the inhibition of shoot branching in *Arabidopsis*. Proc. Natl. Acad. Sci. USA **119**, e2111565119. https://doi.org/10.1073/pnas.2111565119.

Mostofa, M.G., Li, W., Nguyen, K.H., Fujita, M., and Tran, L.S.P. (2018). Strigolactones in plant adaptation to abiotic stresses: An emerging avenue of plant research. Plant Cell Environ. **41**:2227–2243. https://doi.org/10.1111/pce.13364.

Munemasa, S., Hauser, F., Park, J., Waadt, R., Brandt, B., and Schroeder, J.I. (2015). Mechanisms of abscisic acid-mediated control of stomatal aperture. Curr. Opin. Plant Biol. **28**:154–162. https://doi.org/10.1016/j.pbi.2015.10.010.

Munns, R., and Tester, M. (2008). Mechanisms of salinity tolerance. Annu. Rev. Plant Biol. **59**:651–681. https://doi.org/10.1146/annurev.arplant.59.032607.092911.

Omoarelojie, L.O., Kulkarni, M.G., Finnie, J.F., and Van Staden, J. (2019). Strigolactones and their crosstalk with other phytohormones. Ann. Bot. **124**:749–767. https://doi.org/10.1093/aob/mcz100.

Rolland, F., Baena-Gonzalez, E., and Sheen, J. (2006). Sugar sensing and signaling in plants: conserved and novel mechanisms. Annu. Rev. Plant Biol. **57**:675–709. https://doi.org/10.1146/annurev.arplant.57.032905.105441.

Scaffidi, A., Waters, M.T., Ghisalberti, E.L., Dixon, K.W., Flematti, G.R., and Smith, S.M. (2013). Carlactone-independent seedling morphogenesis in *Arabidopsis*. Plant J. **76**:1–9. https://doi.org/10.1111/tpj.12265.

Seto, Y., Sado, A., Asami, K., Hanada, A., Umehara, M., Akiyama, K., and Yamaguchi, S. (2014). Carlactone is an endogenous biosynthetic precursor for strigolactones. Proc. Natl. Acad. Sci. USA **111**:1640–1645. https://doi.org/10.1073/pnas.1314805111.

Shabek, N., Ticchiarelli, F., Mao, H., Hinds, T.R., Leyser, O., and Zheng, N. (2018). Structural plasticity of D3-D14 ubiquitin ligase in strigolactone signalling. Nature **563**:652–656. https://doi.org/10.1038/s41586-018-0743-5.

Shang, Y., and Huang, S. (2020). Engineering Plant Cytochrome P450s for Enhanced Synthesis of Natural Products: Past Achievements and Future Perspectives. Plant Commun. **1**, 100012. https://doi.org/10.1016/j.xplc.2019.100012.

Snowden, K.C., Simkin, A.J., Janssen, B.J., Templeton, K.R., Loucas, H.M., Simons, J.L., Karunairetnam, S., Gleave, A.P., Clark, D.G., and Klee, H.J. (2005). The Decreased apical dominance1/Petunia hybrida CAROTENOID CLEAVAGE DIOXYGENASE8 gene affects branch production and plays a role in leaf senescence, root growth, and flower development. Plant Cell **17**:746–759. https://doi.org/10.1105/tpc.104.027714.

Sorefan, K., Booker, J., Haurogné, K., Goussot, M., Bainbridge, K., Foo, E., Chatfield, S., Ward, S., Beveridge, C., Rameau, C., and Leyser, O. (2003). MAX4 and RMS1 are orthologous dioxygenase-like genes that regulate shoot branching in *Arabidopsis* and pea. Genes Dev. **17**:1469–1474. https://doi.org/10.1101/gad.256603.

Stirnberg, P., van De Sande, K., and Leyser, H.M.O. (2002). MAX1 and MAX2 control shoot lateral branching in *Arabidopsis*. Development **129**:1131–1141.

Stirnberg, P., Furner, I.J., and Ottoline Leyser, H.M. (2007). MAX2 participates in an SCF complex which acts locally at the node to suppress shoot branching. Plant J. **50**:80–94. https://doi.org/10.1111/j.1365-313X.2007.03032.x.

Toh, S., Kamiya, Y., Kawakami, N., Nambara, E., McCourt, P., and Tsuchiya, Y. (2012). Thermoinhibition uncovers a role for strigolactones in *Arabidopsis* seed germination. Plant Cell Physiol. **53**:107–117. https://doi.org/10.1093/pcp/pcr176.

Umehara, M., Cao, M., Akiyama, K., Akatsu, T., Seto, Y., Hanada, A., Li, W., Takeda-Kamiya, N., Morimoto, Y., and Yamaguchi, S. (2015). Structural Requirements of Strigolactones for Shoot Branching Inhibition in Rice and *Arabidopsis*. Plant Cell Physiol. **56**:1059–1072. https://doi.org/10.1093/pcp/pcv028.

Umehara, M., Hanada, A., Yoshida, S., Akiyama, K., Arite, T., Takeda-Kamiya, N., Magome, H., Kamiya, Y., Shirasu, K., Yoneyama, K., et al. (2008). Inhibition of shoot branching by new terpenoid plant hormones. Nature **455**:195–200. https://doi.org/10.1038/nature07272.

Urban, J., Ingwers, M., McGuire, M.A., and Teskey, R.O. (2017). Stomatal conductance increases with rising temperature. Plant Signal. Behav. **12**, e1356534. https://doi.org/10.1080/15592324.2017.1356534.

Wakabayashi, T., Shida, K., Kitano, Y., Takikawa, H., Mizutani, M., and Sugimoto, Y. (2020). CYP722C from Gossypium arboreum catalyzes the conversion of carlactonoic acid to 5-deoxystrigol. Planta **251**:97. https://doi.org/10.1007/s00425-020-03390-6.

Wakabayashi, T., Hamana, M., Mori, A., Akiyama, R., Ueno, K., Osakabe, K., Osakabe, Y., Suzuki, H., Takikawa, H., Mizutani, M., and Sugimoto, Y. (2019). Direct conversion of carlactonoic acid to orobanchol by cytochrome P450 CYP722C in strigolactone biosynthesis. Sci. Adv. **5**, eaax9067. https://doi.org/10.1126/sciadv.aax9067.

Wang, B., Smith, S.M., and Li, J. (2018). Genetic Regulation of Shoot Architecture. Annu. Rev. Plant Biol. **69**:437–468. https://doi.org/10.1146/annurev-arplant-042817-040422.

Wang, L., Wang, B., Jiang, L., Liu, X., Li, X., Lu, Z., Meng, X., Wang, Y., Smith, S.M., and Li, J. (2015). Strigolactone Signaling in *Arabidopsis* Regulates Shoot Development by Targeting D53-Like SMXL Repressor Proteins for Ubiquitination and Degradation. Plant Cell **27**:3128–3142. https://doi.org/10.1105/tpc.15.00605.

Wang, L., Wang, B., Yu, H., Guo, H., Lin, T., Kou, L., Wang, A., Shao, N., Ma, H., Xiong, G., et al. (2020). Transcriptional regulation of strigolactone signalling in *Arabidopsis*. Nature **583**:277–281. https://doi.org/10.1038/s41586-020-2382-x.

Waters, M.T., Gutjahr, C., Bennett, T., and Nelson, D.C. (2017). Strigolactone Signaling and Evolution. Annu. Rev. Plant Biol. **68**:291–322. https://doi.org/10.1146/annurev-arplant-042916-040925.

Waters, M.T., Brewer, P.B., Bussell, J.D., Smith, S.M., and Beveridge, C.A. (2012a). The *Arabidopsis* ortholog of rice DWARF27 acts upstream of MAX1 in the control of plant development by strigolactones. Plant Physiol. **159**:1073–1085. https://doi.org/10.1104/pp.112.196253.

Waters, M.T., Nelson, D.C., Scaffidi, A., Flematti, G.R., Sun, Y.K., Dixon, K.W., and Smith, S.M. (2012b). Specialisation within the DWARF14 protein family confers distinct responses to karrikins and strigolactones in *Arabidopsis*. Development **139**:1285–1295. https://doi.org/10.1242/dev.074567.

Xie, X., Mori, N., Yoneyama, K., Nomura, T., Uchida, K., Yoneyama, K., and Akiyama, K. (2019). Lotuslactone, a non-canonical strigolactone from Lotus japonicus. Phytochemistry **157**:200–205. https://doi.org/10.1016/j.phytochem.2018.10.034.

Yoneyama, K., Mori, N., Sato, T., Yoda, A., Xie, X., Okamoto, M., Iwanaga, M., Ohnishi, T., Nishiwaki, H., Asami, T., et al. (2018). Conversion of carlactone to carlactonoic acid is a conserved function of MAX1 homologs in strigolactone biosynthesis. New Phytol. **218**:1522–1533. https://doi.org/10.1111/nph.15055.

Zhang, X., Ju, H.W., Chung, M.S., Huang, P., Ahn, S.J., and Kim, C.S. (2011). The R-R-type MYB-like transcription factor, AtMYBL, is involved in promoting leaf senescence and modulates an abiotic stress response in *Arabidopsis*. Plant Cell Physiol. **52**:138–148. https://doi.org/10.1093/pcp/pcq180.

Zhang, Y., van Dijk, A.D.J., Scaffidi, A., Flematti, G.R., Hofmann, M., Charnikhova, T., Verstappen, F., Hepworth, J., van der Krol, S., Leyser, O., et al. (2014). Rice cytochrome P450 MAX1 homologs catalyze distinct steps in strigolactone biosynthesis. Nat. Chem. Biol. **10**:1028–1033. https://doi.org/10.1038/nchembio.1660.

Plant Communications, Volume *4*

Supplemental information

AtMYBS1 negatively regulates heat tolerance by directly repressing the expression of *MAX1* required for strigolactone biosynthesis in *Arabidopsis*

Xiang Li, Jianhua Lu, Xuling Zhu, Yanqi Dong, Yanli Liu, Shanshan Chu, Erhui Xiong, Xu Zheng, and Yongqing Jiao

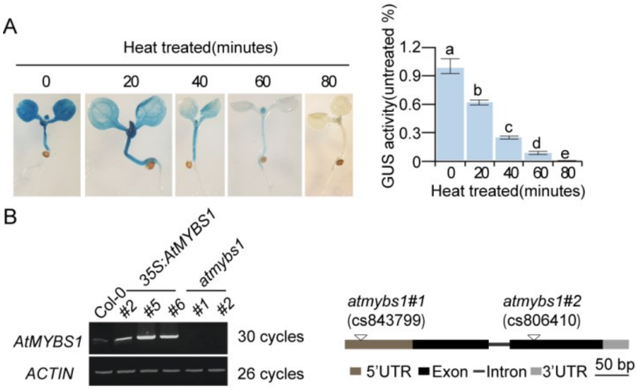

Supplemental Figure 1. Expression pattern of *pAtMYBS1*:*GUS* in response to heat stress and molecular validation of *AtMYBS1* overexpression lines and loss-of-function mutants.
(A) The expression pattern of *pAtMYBS1:GUS* in response to heat stress. Seven-day-old seedlings of *pAtMYBS1:GUS*-positive homozygous transgenic plants grown in half-strength MS medium in a greenhouse (23 °C, 70% humidity, 16 h light/8 h dark cycle) were subjected to heat treatment at 40 °C for the indicated times. Then, GUS staining assays were performed to monitor *AtMYBS1* promoter activity under heat stress conditions. Three independent biological replicates were performed (n>5 for each replicate). The data are the means ± SD. Different letters on error bars indicate significant differences at $P<0.05$, Tukey's *t* test. **(B)** Confirmation of *AtMYBS1* overexpression lines and loss-of-function mutants. Semiquantitative RT-PCRs were performed to measure *AtMYBS1* expression in *AtMYBS1*-overexpressing plants and *atmybs1* mutants. *ACTIN* was used as an internal control. The brown box represents the 5'UTR. The black boxes represent exons, and the black line represents introns. The gray box represents the 3'UTR. Two T-DNA insertion sites are indicated by inverted triangles.

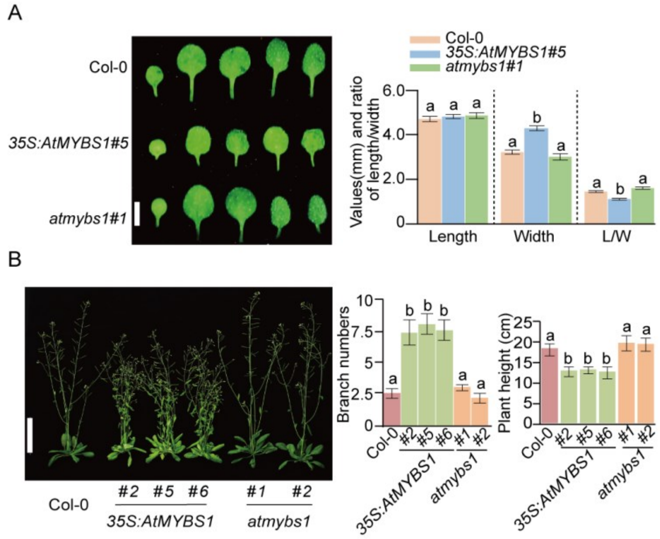

Supplemental Figure 2. Phenotypes of *AtMYBS1*-overexpressing plants and *atmybs1* mutants.
(A) Leaf shape of *AtMYBS1*-overexpressing plants and *atmybs1* mutants. Leaves of 16-day-old plants of Col-0, *35S:AtMYBS1-5* and *atmybs1-1* were collected and photographed; meanwhile, their sizes were measured and documented (n>12 in each sample). L/W: length/width. The data shown are the means ± SD. Different letters on error bars indicate significant differences at P<0.05, Tukey's *t* test. Scale bar=5 mm. **(B)** Plant morphologies of *AtMYBS1*-overexpressing plants and *atmybs1* mutants. Branch numbers and plant height from 40-day-old plants of Col-0, *AtMYBS1*-overexpressing lines (*35S:AtMYBS1-2*, *-5* and *-6*) and *atmybs1* (*atmybs1-1* and *-2*) mutants were observed and analyzed (n>10). The data are the means ± SDs. Different letters on error bars indicate significant differences at P<0.05, Tukey's *t* test. Scale bar=5 cm.

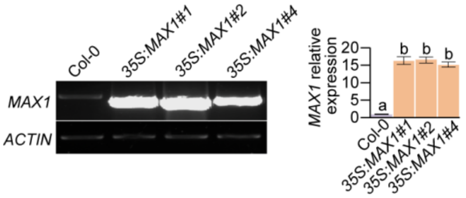

Supplemental Figure 3. The expression of *MAX1* in *MAX1*-overexpressing transgenic lines.
The *35S:MAX1* construct (*pMDC85* vector) was constructed and transformed into wild-type Col-0 plants, and homozygous single copy T-DNA insertion lines were screened and obtained. Seeds of the *35S:MAX1#1, #2,* and *#4* transgenic lines were sown on half-strength MS medium and grown at 23 °C in the greenhouse (16 hours light/8 hours dark, 70% humidity). The 14-day-old plants were harvested for RNA extraction, and semiquantitative RT–PCR was employed to measure *MAX1* expression (left). qRT–PCR was also performed to confirm *MAX1* expression levels (right). Three independent biological replicates were performed. The data are the means ± SDs. Different letters on error bars indicate significant differences at $P<0.05$, Tukey's *t* test.

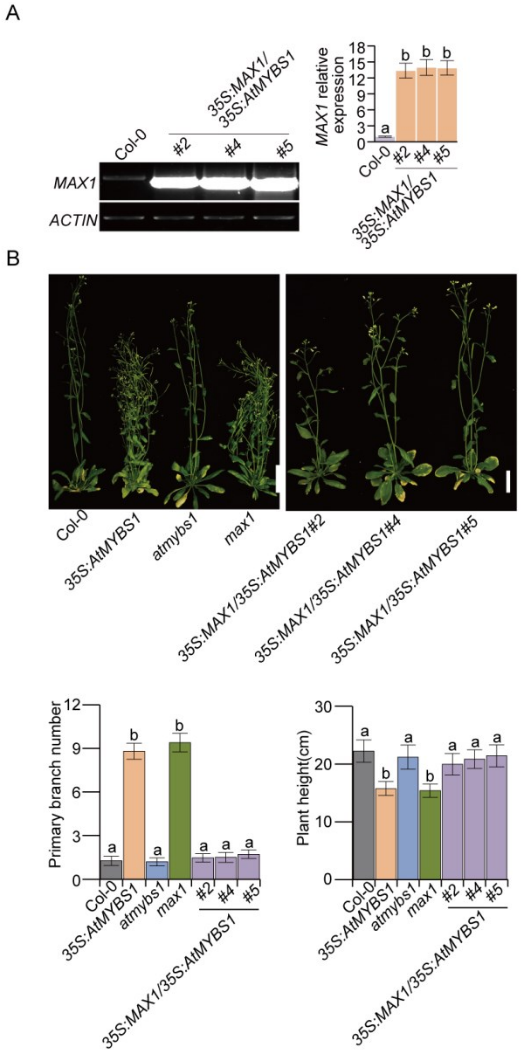

Supplemental Figure 4. Plant morphologies of *35S:MAX1*/*35S:AtMYBS1* plants.
(A) *MAX1* expression levels in *35S:MAX1/35S:AtMYBS1* plants. The *35S:MAX1* (*pMDC85* vector) construct was transformed into *35S:AtMYBS1-5* plants. T_3 generation plants were screened for homozygous single copy T-DNA insertion lines. Seeds from homozygous lines were sown on half-strength MS medium and grown at 23 °C in the greenhouse (16 hours light/8 hours dark, 70% humidity). The 12-day-old plants were harvested for RNA extraction, and semiquantitative RT–PCR was employed to measure *MAX1* expression (left). qRT–PCR was also performed to confirm the *MAX1* expression level (right). Three independent biological replicates were conducted in the qRT–PCR experiments. The data are the means ± SDs. Different letters on error bars indicate significant differences at P<0.05, Tukey's *t* test. **(B)** Phenotypes of Col-0, *35S:AtMYBS1-5*, *atmybs1-1*, *max1-1* and *35S:MAX1/35S:AtMYBS1-5* transgenic lines. Six-week-old plants of Col-0, *35S:AtMYBS1-5*, *atmybs1-1*, *max1-1* and *35S:MAX1/35S:AtMYBS1-5* transgenic lines (*35S:MAX1/35S:AtMYBS1#2, #4, #5*) grown in a greenhouse were collected and photographed. Their branch numbers and plant height were measured and statistically analyzed (n>10). The data are the means ± SDs. Different letters on error bars indicate significant differences at P<0.05, Tukey's *t* test. Scale bar=4 cm (left), 2 cm (right).

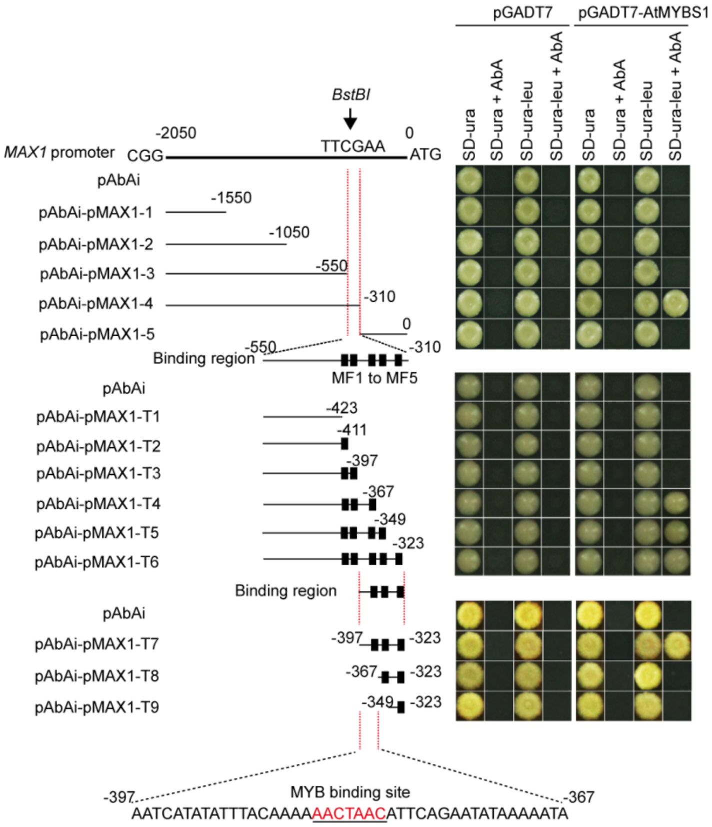

Supplemental Figure 5. The interaction between AtMYBS1 and the *MAX1* promoter detected by yeast one-hybrid assays.

Yeast one-hybrid assays were performed to investigate the interaction between AtMYBS1 and the *MAX1* promoter. The bait vectors (*pAbAi-pMAX1-T1* to *pAbAi-pMAX1-T9*) and the prey vector *pGADT7-AtMYBS1* were constructed and transformed into yeast strain Y1HGold and then plated onto different nutrient-deficient media to examine their interactions. The empty vectors *pAbAi* and *pGADT7* were used as negative controls. All of the solid lines in the picture indicate truncated *MAX1* promoter regions incorporated into *pAbAi*. Numbers indicate exact positions in the *MAX1* promoter. The black boxes represent predictive motifs. The red dotted lines indicate regions to which *AtMYBS1* directly binds according to the yeast one-hybrid results. The underlined red letters indicate MYB-binding sites. Each bait and prey vector was validated by PCR amplification after transformation into the yeast Y1HGold strain. All hybrid assays were repeated at least three times.

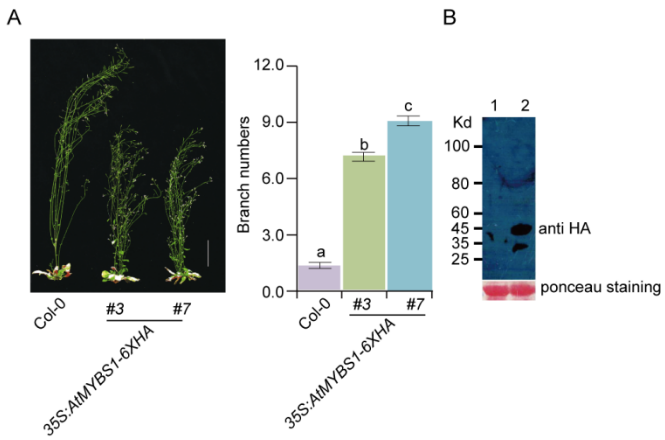

Supplemental Figure 6. Functional confirmation of AtMYBS1-6×HA proteins.
(A) Plant morphologies of HA-tagged *AtMYBS1*-overexpressing transgenic plants. Seven-week-old *35S:AtMYBS1-6×HA* transgenic plants were collected, and their branch numbers were counted (n>15). The data are the means ± SD. Different letters on error bars indicate significant differences at P<0.05, Tukey's *t* test. Scale bar=4 cm. **(B)** Expression of AtMYBS1-6×HA in *35S:AtMYBS1-6×HA* transgenic lines (*35S:AtMYBS1-6×HA*-3 and *35S:AtMYBS1-6×HA*-7) by western blotting. Total proteins were extracted from 12-day-old seedlings of Col-0 and *35S:AtMYBS1-6xHA*-3 and *35S:AtMYBS1-6×HA*-7 transgenic plants. HA antibody was used to detect AtMYBS1-HA expression. Ponceau staining was used as an internal control. The number 1 indicates Col-0; 2 indicates 3*5S:AtMYBS1-6×HA*-7.

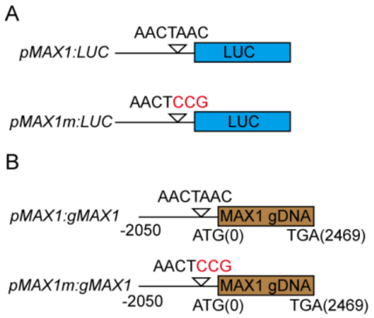

Supplemental Figure 7. Schematic illustrations of constructed vectors.
(**A**) Schematic illustrations of *pMAX1:LUC* and *pMAX1m:LUC* vectors. The black solid lines indicate the *MAX1* promoter. The reverse triangles represent MYB-binding sites. The red letters represent mutated sequences (AAC were changed into CCG). The blue boxes represent luciferase coding sequences. (**B**) Schematic illustrations of *pMAX1:gMAX1* and *pMAX1m:gMAX1* vectors. The black lines indicate the *MAX1* promoter. The inverted triangles represent MYB-binding sites. The red letters indicate mutated nucleotides (AAC were changed into CCG). The brown boxes indicate the *MAX1* genomic sequence after the start codon. Numbers indicate locations corresponding to the *MAX1* genomic sequence.

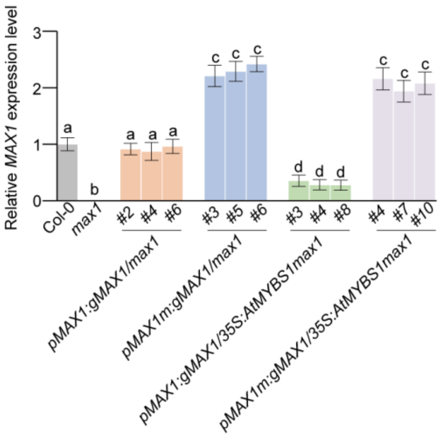

Supplemental Figure 8. ***MAX1*** **expression in** ***pMAX1:gMAX1*** **and** ***pMAX1m:gMAX1*** **transgenic plants in the** ***max1*** **and** ***35S:AtMYBS1max1*** **backgrounds.**

The expression of *MAX1* was measured by qRT–PCR in Col-0, *max1-1*, *pMAX1:gMAX1* transgenic plants in the *max1* background (*pMAX1:gMAX1/max1* #2, #4 and #6), *pMAX1m:gMAX1* transgenic plants in the *max1* background (*pMAX1m:gMAX1/max1* #3, #5 and #6), *pMAX1:gMAX1* transgenic plants in *35S:AtMYBS1max1* (*pMAX1:gMAX1/35S:AtMYBS1max* #3, #4 and #8) and *pMAX1m:gMAX1* transgenic plants in the *35S:AtMYBS1max1* (*pMAX1m:gMAX1/35S:AtMYBS1max1* #4, #7 and #10) background. The *35S:AtMYBS1max1* plants were generated by overexpressing *AtMYBS1* under the 35S promoter in the *max1-1* background. Twelve-day-old seedlings grown on half-strength MS (Murashige and Skoog) medium were harvested for RNA extraction and qRT–PCR analysis to measure *MAX1* expression. Three independent biological replicates were carried out. The data are the means ± SDs. Different letters on error bars indicate significant differences at P<0.05, Tukey's *t* test.

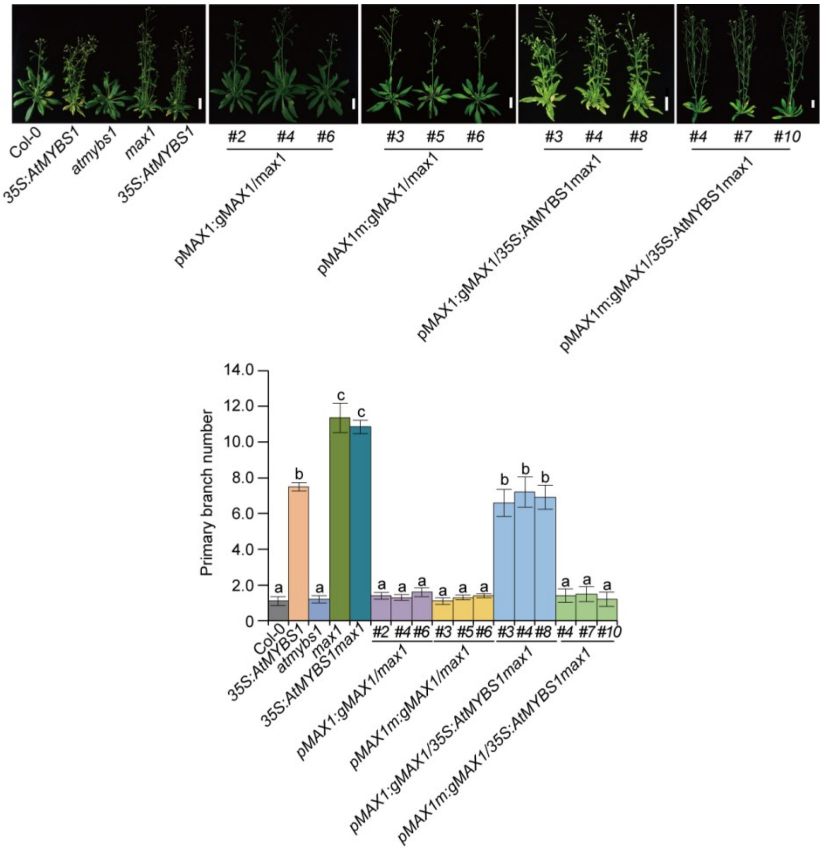

Supplemental Figure 9. Plant morphologies of *pMAX1:gMAX1* and *pMAX1m:gMAX1* transgenic plants in the *max1* and *35S:AtMYBS1max1* backgrounds.

Plant morphologies of Col-0, *35S:AtMYBS1-5, atmybs1-1, max1-1, 35S:AtMYBS1max1-1* #1, *pMAX1:gMAX1* transgenic plants in the *max1* background (*pMAX1:gMAX1/max1* #2, #4 and #6), *pMAX1m:gMAX1* transgenic plants in the *max1* background (*pMAX1m:gMAX1/max1* #3, #5 and #6), *pMAX1:gMAX1* transgenic plants in *35S:AtMYBS1max1* (*pMAX1:gMAX1/35S:AtMYBS1max1* #3, #4 and #8) and *pMAX1m:gMAX1* transgenic plants in the *35S:AtMYBS1max1* (*pMAX1m:gMAX1/35S:AtMYBS1max1* #4, #7 and #10) background. The *35S:AtMYBS1max1* plants were generated by overexpressing *AtMYBS1* under the 35S promoter in the *max1-1* background. All of the above plants were grown in a greenhouse (23 °C, 70% humidity, 16 h light/8 h dark cycle). After 6 weeks, when all the plants had bolted and flowered, different plant samples were harvested, and the number of branches was counted (n>10). The data are the means ± SD. Different letters on error bars indicate significant differences at $P<0.05$, Tukey's *t* test.

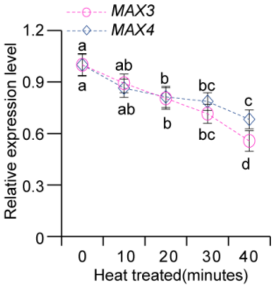

Supplemental Figure 10. ***MAX3*** **and** ***MAX4*** **expression in response to heat stress.**
The wild-type Col-0 seeds sown on half-strength MS medium were grown at 23 °C in the greenhouse (16 hours light/8 hours dark, 70% humidity). The 12-day-old seedlings (two true leaves developed) were treated at 40 °C in a climate chamber (40 °C, 60% humidity, 16 h light/8 h dark cycle) with the time indicated. RNA was extracted from the treated plants after heat treatments, and quantitative RT–PCR was carried out to measure *MAX3* and *MAX4* expression. Three independent biological replications were performed. The data are the means± SDs. Different letters on error bars indicate significant differences at $P<0.05$, Tukey's *t* test.

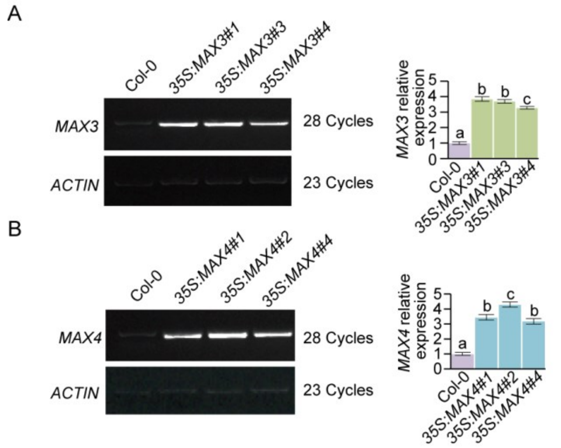

Supplemental Figure 11. Validation of *MAX3* and *MAX4* expression in *35S:MAX3* and *35S:MAX4* plants.

The *35S:MAX3* (*pJL12*) and *35S:MAX4* (*pJL12*) constructs were generated and transformed into wild-type Col-0 plants. Homozygous single copy T-DNA insertion lines were screened and obtained. The seeds of homozygous lines were sown on half-strength MS medium and grown at 23 °C in a greenhouse (16 hours light/8 hours dark, 70% humidity). The 14-day-old plants were harvested for RNA extraction. Subsequent semiquantitative RT–PCR was employed to measure *MAX3* and *MAX4* expression (left). qRT–PCR was also performed to confirm *MAX3* and *MAX4* expression levels **(**right**)**. Three independent biological replicates were conducted. The data are the means ± SDs. Different letters on error bars indicate significant differences at $P<0.05$, Tukey's *t* test.

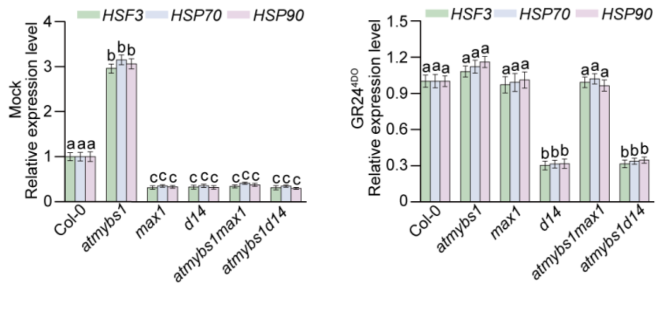

Supplemental Figure 12. Expression of *HSF3, HSP70* and *HSP90* in *max1* mutants, *atmybs1max1* double mutants and *atmybs1d11* double mutants without or with $GR24^{4DO}$ application.
Expression levels of *HSF3*, *HSP70* and *HSP90* were measured by qRT–PCR in Col-0 and atmybs1-1, *max1-1*, *d14-1*, *atmybs1-1max1-1* and *atmybs1-1d14-1* double mutants without or with $GR24^{4DO}$ application. The 14-day-old seedlings grown on half-strength MS medium were collected for RNA extraction. Subsequently, qRT–PCR was performed to measure *HSF3*, *HSP70* and *HSP90* expression. Three independent biological replicates were carried out. The data are the means ± SDs. Different letters on error bars indicate significant differences at $P<0.05$, Tukey's *t* test.

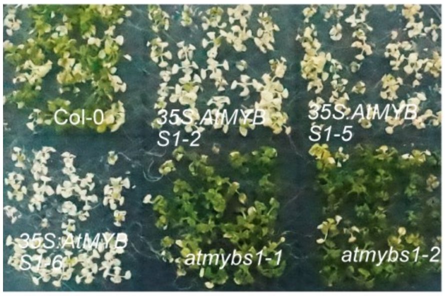

Figure 1B

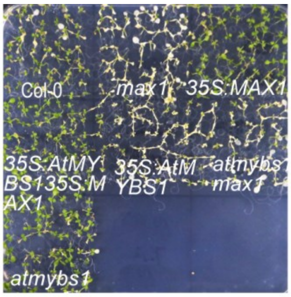

Figure 2C

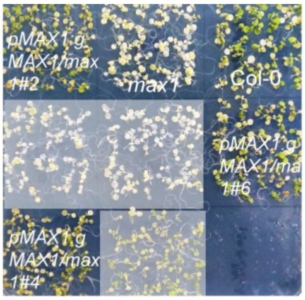

Figure 3D

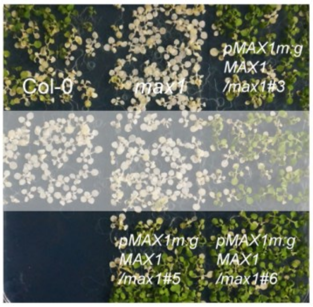

Figure 3D

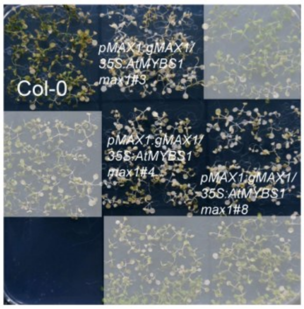

Figure 3E

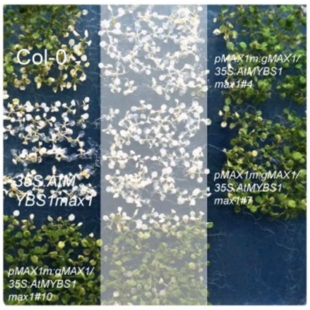

Figure 3E

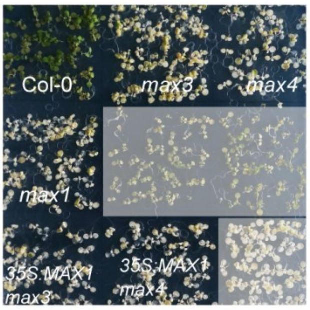

Figure 4A

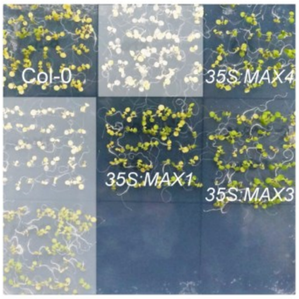

Figure 4A

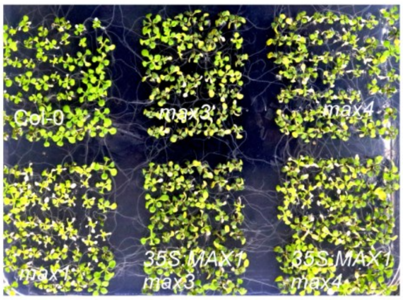

Figure 4B

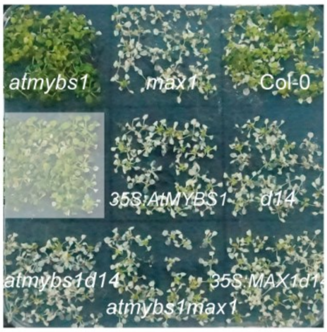

Figure 4C

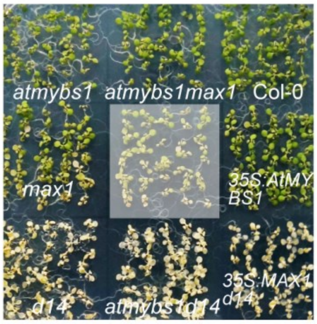

Figure 4C

Supplemental Figure 13. Original photographs for related pictures in this study.
Pictures of the original whole petri dishes used in this study are shown. The samples unrelated to this study are covered by white boxes. The samples used in this study were labeled as indicated.

Supplemental Table 1. Primers used in vector construction and gene expression analyses.

name	sequence	usage
pJL12-AtMYBS1-F	TCTGATCAAGAGACAGATGGAGAGTGTGGTGGCAACATGGAGC	*35S:AtMYBS1*(*pJL12*)
pJL12-AtMYBS1-R	GCTCTAGAACTAGTGTCAGTGCATTGTCGACGGAG	
pAtMYBS1-GUS-F	CAAAAAAGCAGGCTTTGACTTTAGGTC CACCGGACATTTTCTTAAAT	*pAtMYBS1:GUS* (*pKGWFS7*)
pAtMYBS1-GUS-R	AAAGCTGGGTCTAGAGACTTTAGGTC TCGATTGCTTCAATCTTTTC	
pMDC85-MAX1-F	TTCTAGAGTCGAGGTCC ATGAAGACGCAACATCAATG	*35S:MAX1*(*pMDC85*)
pMDC85-MAX1-R	CGGTAGAAAAAATGA TCAGAATCTTTTGATGGTTCTGA	
pGADT7-AtMYBS1-F	ATGGCCATGGAGGCCAGTGAATTC ATGGAGAGTGTGGTGGCAAC	*pGADT7-AtMYB1*
pGADT7-AtMYBS1-R	GGATCCATCGAGCTCGAGCTGCA TCAGTGCATTGTCGACGGAG	
pAbAi-pMAX1-1-F	GCTTGAATTCGAGCT CGGTGATAAACTAATCCACCA	*pAbAi-pMAX1-1*
pAbAi-pMAX1-1-R	AGCACATGCCTCGAGG TTCCTTCCTCCAACAAGATG	
pAbAi-pMAX1-2-F	GCTTGAATTCGAGCT CGGTGATAAACTAATCCACCA	*pAbAi-pMAX1-2*
pAbAi-pMAX1-2-R	AGCACATGCCTCGAGG GGGAGAGACGAAATTTCATC	
pAbAi-pMAX1-3-F	GCTTGAATTCGAGCT CGGTGATAAACTAATCCACCA	*pAbAi-pMAX1-3*
pAbAi-pMAX1-3-R	AGCACATGCCTCGAGG GCTTCTACTTTTGGTCTCCT	
pAbAi-pMAX1-4-F	GCTTGAATTCGAGCT CGGTGATAAACTAATCCACA	*pAbAi-pMAX1-4*
pAbAi-pMAX1-4-R	AGCACATGCCTCGAGG GAAACTATTTAACTATATT	
pAbAi-pMAX1-5-F	GCTTGAATTCGAGCT GAATTAAACACTAAATAATTAAATGTTGAC	*pAbAi-pMAX1-5*
pAbAi-pMAX1-5-R	AGCACATGCCTCGAGG CTCTCTAACCTCTAAAGTTC	
pAbAi-pMAX1-T1-F	GCTTGAATTCGAGCT CTCTCTAACCTCTAAAGTTC	*pAbAi-pMAX1-T1*
pAbAi-pMAX1-T1-R	AGCACATGCCTCGAGG TACTATCTTTTGAGTATGGG	
pAbAi-pMAX1-T2-F	GCTTGAATTCGAGCT GTGGTTTACTCCAATTGACGG	*pAbAi-pMAX1-T2*
pAbAi-pMAX1-T2-R	AGCACATGCCTCGAGG AAATAAACAGAATACTATCT	
pAbAi-pMAX1-T3-F	GCTTGAATTCGAGCT GTGGTTTACTCCAATTGACGG	*pAbAi-pMAX1-T3*
pAbAi-pMAX1-T3-R	AGCACATGCCTCGAGG AAATATATGATTATAAATAA	
pAbAi-pMAX1-T4-F	GCTTGAATTCGAGCT GTGGTTTACTCCAATTGACGGT	*pAbAi-pMAX1-T4*
pAbAi-pMAX1-T4-R	AGCACATGCCTCGAGG ATTTTTATATTCTGAATGTT	
pAbAi-pMAX1-T5-F	GCTTGAATTCGAGCT GTGGTTTACTCCAATTGACGGTAAA	*pAbAi-pMAX1-T5*
pAbAi-pMAX1-T5-R	AGCACATGCCTCGAGG AAATTTACATATTCTAATAT	
pAbAi-pMAX1-T6-F	GCTTGAATTCGAGCT GTGGTTTACTCCAATTGACGG	*pAbAi-pMAX1-T6*
pAbAi-pMAX1-T6-R	AGCACATGCCTCGAGG ATATTTAATACTCCTCTATG	
pAbAi-pMAX1-T7-F	GCTTGAATTCGAGCT TGGTTTACTCCAATTGACGGTAA	*pAbAi-pMAX1-T7*
pAbAi-pMAX1-T7-R	AGCACATGCCTCGAGG ATATTTAATACTCCTCTATG	
pAbAi-pMAX1-T8-F	GCTTGAATTCGAGCT TGACAAGTAGAGGCACTTGATAGTAGTGT	*pAbAi-pMAX1-T8*
pAbAi-pMAX1-T8-R	AGCACATGCCTCGAGG ATATTTAATACTCCTCTATG	
pAbAi-pMAX1-T9-F	GCTTGAATTCGAGCT GATAGTAGTGTAACAAAAGT	*pAbAi-pMAX1-T9*
pAbAi-pMAX1-T9-R	AGCACATGCCTCGAGG ATATTTAATACTCCTCTATG	
pAbAi-pMAX1-T10	GCTTGAATTCGAGCT ACAAAAAACTAACATTCAGAATATAAAAAT CCTCGAGGCATGTGCT	*pAbAi-pMAX1-T10*
pAbAi-pMAX1-MD	GCTTGAATTCGAGCT ACAAAAATTCAGAATATAAAAAT CCTCGAGGCATGTGCT	*pAbAi-pMAX1-MD*

pAbAi-pMAX1-MP	GCTTGAATTCGAGCT ACAAAAAACTCCGATTCAGAATATAAAAT CCTCGAGGCATGTGCT	*pAbAi-pMAX1-MP*
pJL12-AtMYBS1-6×HA-F	GATCTGATCAAGAGACAG ATGGAGAGTGTGGTGGCAACATGGAGC	*35S:AtMYBS1*(*pJL12-6×HA*)
pJL12-AtMYBS1-6×HA-R	GCCCTTGCTCACCATGGA GTGCATTGTCGACGGAG	
pMAX1-pGreenII0800-F	TCGACGGTATCGATA **C**GGTGATAAACTAATCCACCAA	*pMAX1-pGreenII0800*
pMAX1-pGreenII0800-R	TAGAACTAGTGGATC CTCTCTAACCTCTAAAGTTCTCTCTT	
pMAX1m-pGreenII0800-F1	TCGACGGTATCGATA **C**GGTGATAAACTAATCCAC	*pMAX1m-pGreenII0800*
pMAX1m-pGreenII0800-R1	CTGAATCGGAGTTTTTTGTAAAT	
pMAX1m-pGreenII0800-F2	TATTTACAAAAAACTCCGATTCAGAAT	
pMAX1m-pGreenII0800-R2	TAGAACTAGTGGATC CTCTCTAACCTCTAAAGTTCTCTCTT	
pMAX1-gMAX1-F	TATGACCATGATTACG **C**GGTGATAAACTAATCCACCAA	*pMAX1:gMAX1(p1300)*
pMAX1-gMAX1-R	AGGTCGACTCTAGAG TCAGAATCTTTTGATGGTTCTGAG	
pMAX1m-gMAX1-F1	TATGACCATGATTACG **C**GGTGATAAACTAATCCACCAA	*pMAX1m:gMAX1(p1300)*
pMAX1m-gMAX1-R1	CTGAATCGGAGTTTTTTGTAAAT	
pMAX1m-gMAX1-F2	TATTTACAAAAAACTCCGATTCAGAAT	
pMAX1m-gMAX1-R2	AGGTCGACTCTAGAG TCAGAATCTTTTGATGGTTCTG	
pJL12-MAX3-F	TCTGATCAAGAGACAG ATGTCTCTCCCTATCCCGCCGAA	*35S:MAX3*(*pJL12*)
pJL12-MAX3-R	GCTCTAGAACTAGTG TTATTTGTGTGAAAGGTGAAAAG	
pJL12-MAX4-F	TCTGATCAAGAGACAG ATGGCTTCTTTGATCACAACCAA	*35S:MAX4*(*pJL12*)
pJL12-MAX4-R	GCTCTAGAACTAGTG TTAATCTTTGGGGATCCAGCAAC	
MAX1-qPCR-F	ATGAAGACGCAACATCAATG	
MAX1-qPCR-R	ATCTTTGAACTTCTTTATCC	
MAX2-qPCR-F	ATGGCTTCCACTACTCTCTC	
MAX2-qPCR-R	GTGTGTAGACGTTTAGAGAC	
MAX3-qPCR-F	ATGTCTCTCCCTATCCCGCC	
MAX3-qPCR-R	GGTTCGATAGTCTCGGAACG	
MAX4-qPCR-F	ATGGCTTCTTTGATCACAAC	
MAX4-qPCR-R	TTGTTGTACACTTGTCCACG	
AtMYBS1-qPCR-F	ATGGAGAGTGTGGTGGCAAC	
AtMYBS1-qPCR-R	CCTTTGCGGTGATGATAACG	
ACTIN-qPCR-F	CTCAGCACCTTCCAACAGATGTGGA	
ACTIN-qPCR-R	CCAAAAAAATGAACCAAGGACCAAA	qRT-PCR
HSF3- qPCR-F	ATGAGCCCAAAAAAAGATGCTGTTT	
HSF3- qPCR-R	ATCCCAAACTACGAAGCTAG	
HSP90-qPCR-F	ATGAGGAAGAGGACGCTCGT	
HSP90-qPCR-R	CTCAAACTTCTCCGCGTTAC	
HSP70-qPCR-F	ATGGCGGGTAAAGGTGAAGG	
HSP70-qPCR-R	GAACAGAGGGATCACTGTAT	
R1f	CTCTCTTCTCCACTCTTGCAG	
R1r	CCAGCTCCTAACCCCTTCAC	
R2f	GGTTAAATTGATGAGGTCTC	
R2r	TCAATACATGTAATGCAGAT	
R3f	GTAACTAGATGAAAGAAAAGAA	

R3r	AAAGGAGAAATTTGATCATT	
R4f	TCAAACATCAAAAGGACCCC	
R4r	TTTTTTCTTGTCAACATTTA	
R5f	ATTTACAAAAAACTAACATTCA	
R5r	CGCAAGAAGACAACATGCGT	
R6f	TTAGCAATATTACTCTCTAGTAGA	
R6r	AAAGTTCTCTCTTCTTTAGT	
atmybs1-1-LP	TTATTATTTGCCGCAGTTTCG	*atmybs1-1* genotyping
atmybs1-1-RP	TTACCTCGTTATCATCACCGC	
atmybs1-2-LP	CAAGCTCCTTATCGCGAAAG	*atmybs1-2* genotyping
atmybs1-2-RP	ACCACACTCTCCATTCGATTG	
LB1	GCCTTTTCAGAAATGGATAAATAGCCTTGCTTCC	Universal primer for genotyping

Note: The underlined sequences are adaptors in destiny vectors used in homologous recombination. Brackets indicate destiny vectors that the sequences are ultimately integrated in.
